# An Extended, Boolean Model of the Septation Initiation Network in *S*.*Pombe* Provides Insights into Its Regulation

**DOI:** 10.1371/journal.pone.0134214

**Published:** 2015-08-05

**Authors:** Anastasia Chasapi, Paulina Wachowicz, Anne Niknejad, Philippe Collin, Andrea Krapp, Elena Cano, Viesturs Simanis, Ioannis Xenarios

**Affiliations:** 1 Vital-IT Group, Swiss Institute of Bioinformatics (SIB), Lausanne, Switzerland; 2 Cell cycle control laboratory, Ecole Polytechnique Fédérale de Lausanne (EPFL), SV-ISREC, Lausanne, Switzerland; 3 Swiss-Prot Group, Swiss Institute of Bioinformatics (SIB), Geneva, Switzerland; Cancer Research UK London Research Institute, UNITED KINGDOM

## Abstract

Cytokinesis in fission yeast is controlled by the Septation Initiation Network (SIN), a protein kinase signaling network using the spindle pole body as scaffold. In order to describe the qualitative behavior of the system and predict unknown mutant behaviors we decided to adopt a Boolean modeling approach. In this paper, we report the construction of an extended, Boolean model of the SIN, comprising most SIN components and regulators as individual, experimentally testable nodes. The model uses CDK activity levels as control nodes for the simulation of SIN related events in different stages of the cell cycle. The model was optimized using single knock-out experiments of known phenotypic effect as a training set, and was able to correctly predict a double knock-out test set. Moreover, the model has made *in silico* predictions that have been validated *in vivo*, providing new insights into the regulation and hierarchical organization of the SIN.

## Introduction

*Schizosaccharomyces pombe*, commonly referred as fission yeast, has long been used as a model organism for the study of conserved, essential functions in the eukaryotic cell. It has proved highly informative in the study of the cell cycle, particularly the control of the G2/M transition. Like many somatic higher eukaryotic cells, it divides by binary fission. Cytokinesis in fission yeast is controlled by the Septation Initiation Network (SIN), a protein kinase signaling network, which uses the spindle pole body (SPB; the functional counterpart of the centrosome in yeast), as a scaffold from which to initiate signaling. Elements of the SIN signaling architecture have been conserved throughout evolution. In *Saccharomyces cerevisiae* the corresponding pathway is known as the mitotic exit network (MEN), and controls both cytokinesis and mitotic exit. In higher eukaryotes the equivalent signaling network is the hippo pathway, which regulates cell growth and proliferation [1,2].

The SIN comprises a group of protein kinases and their regulators that induce cytokinesis when CDK activity drops in anaphase [3–5]. Signaling failure results in multinucleated cells, as cytokinesis fails while growth and the nuclear cycle continue [6], which is referred to as the SIN phenotype. Failure to turn off SIN signaling produces multiseptated cells that remain uncleaved and contain one or two nuclei [7]. Ppc89p, Cdc11p and Sid4p form the scaffold upon which signaling proteins are assembled at the SPB [8–11]. SIN signaling requires the action of three kinase complexes. The association properties of SIN proteins with the SPB differ in early and late mitosis (see [12], and references for each protein cited below). The kinase Cdc7p associates with the signaling GTPase Spg1p [13,14], Sid1p associates with its regulatory subunit Cdc14p [4,15] and the kinase Sid2p associates with its regulator Mob1p [16–18]. Association of the SIN kinase modules with the SPB during mitosis is considered to indicate that the kinase in question is active (reviewed by [19,20]). The nucleotide status of Spg1p is regulated by a bipartite GAP, composed of a catalytic subunit (Cdc16p), which interacts with Spg1p in the context of a scaffold, Byr4p [21,22]. Etd1p regulates the nucleotide status of Spg1p, perhaps by modulating Rho1p signaling [23–26]. Plo1p acts upstream of the SIN [27,28] and coordinates SIN activity with other mitotic events. The SIN controls many aspects of cytokinesis including the assembly of the contractile ring and synthesis of the division septum [29].

Our goal is to describe the qualitative behavior of the system, investigate the role of each SIN regulator and potentially predict unknown mutant behaviors. Towards this end we adopted a Boolean modeling approach. The choice of qualitative modeling was based on their suitability to simulate systems with restricted kinetic data, as well as their computational efficiency, that permits large numbers of *in silico* experiments even in networks with hundreds of nodes.

Computational models find their origins in engineering science, and have proved to be useful tools with which to analyze complex biological systems (for example [30–32]). The different types of modeling techniques can vary from qualitative Boolean models, to quantitative kinetic-based models; which of them is chosen depends on the type and amount of knowledge and experimental data available for the specific system, as well as the size of the network [33,34].

The cell cycles of fission and budding yeast have long been popular fields of research and several modeling strategies have been employed to understand them [30,35–40]. Models focused on the fission yeast SIN have already been generated by Csikasz-Nagy et al. (2007) and Bajpai et al. (2013) [41,42]. In the study by Csikasz-Nagy *et al*. the timing of septation in wild type and mutant cells was described using a minimal, continuous model. The SIN components were treated as two groups, the “Top of SIN” and “Bottom of SIN”, with Sid1p localization to the SPB being the pivotal event that differentiates the two groups [42]. In the subsequent model [41], the asymmetric distribution of molecules at the SPBs was analyzed using a simple, non-linear model of two antagonistic molecules. The model was also extended to incorporate key regulators of the SIN [41].

In this work, we present an extended, Boolean model of the SIN, comprising most known SIN components and regulators as individual, experimentally testable nodes. The Boolean framework allows us to perform *in silico* knock-out and “constant activation” experiments for every combination of molecules present in the model, and to assess phenotypic predictions that could be subsequently validated experimentally. Our model provided useful insights for several aspects of SIN regulation such as the role of Fin1p, the inhibitory function of Nuc2p in interphase, as well as an *in silico*, counter-intuitive, double mutant phenotypic prediction. The model predicted that Sid4p mutant cells would septate if they express Cdc7p in high levels. The prediction has been experimentally confirmed. This work serves as a good example of the use of qualitative modeling in hypotheses generation and prediction of experimental outcomes in otherwise complicated and long experiments.

## Results

### Model construction through expert biocuration

**Fig 1:**
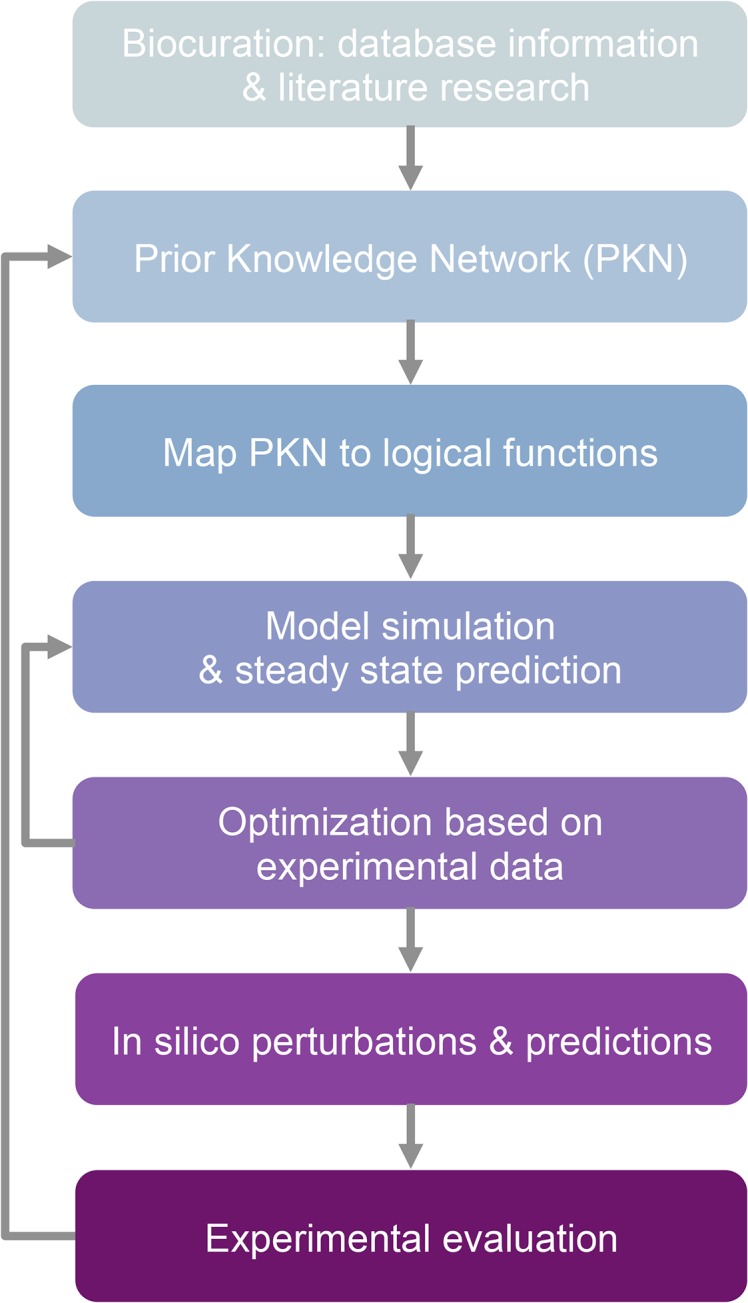
Model construction and optimization workflow. The Prior Knowledge Network (PKN) is constructed after collecting relevant information from various sources, including network databases and literature. The PKN is translated into logical functions, describing the regulatory relations among gene products. The logical model is simulated under the preferred conditions, resulting in one or more steady states, where all logical rules are satisfied. The model goes then through an optimization procedure, where the goal is to fit the resulting steady states with available experimental data by altering regulatory rules. The optimization typically includes removing outdated / low confidence links, adjusting their representation and adding new regulatory rules. The process is iterated until the simulation fits the available data. The model can then be used as a predictive tool, by performing *in silico* perturbations. Validation of the predictions can lead to discovery of missing regulatory links that are then added to the PKN.

An overview of the workflow used for the model construction, optimization and use is presented in Fig 1. For the gene regulatory network construction of the SIN we chose an expert biocuration approach [43,44], taking advantage of the long-term expertise in the Swiss-Prot group. Experimentally determined interactions specific to the SIN, were retrieved, structured, curated and annotated from the literature and from available knowledge databases (for example Pubmed, iHOP, UniProtKB/Swiss-Prot, ChEBI). To generate the model, we started by adding the main SIN signaling regulators such as the GTPase Spg1p, its effector kinase Cdc7p and the GAP Byr4p and Cdc16p [13,21,45]. We then added the SPB scaffold for the SIN, which is comprised of Ppc89p, Sid4p and Cdc11p [10,45]. Subsequently, additional regulators were added to this core unit, to complete a first working model. The collected knowledge was stored in a structure formed of pairwise interactions and regulations that include information about participating components, the origin of publications (PMID), the evidence used to evaluate the interaction was mentioned and a confidence assessment as an evidence tag from the biocurator (a full interaction table provided in S1 Table).

**Fig 2:**
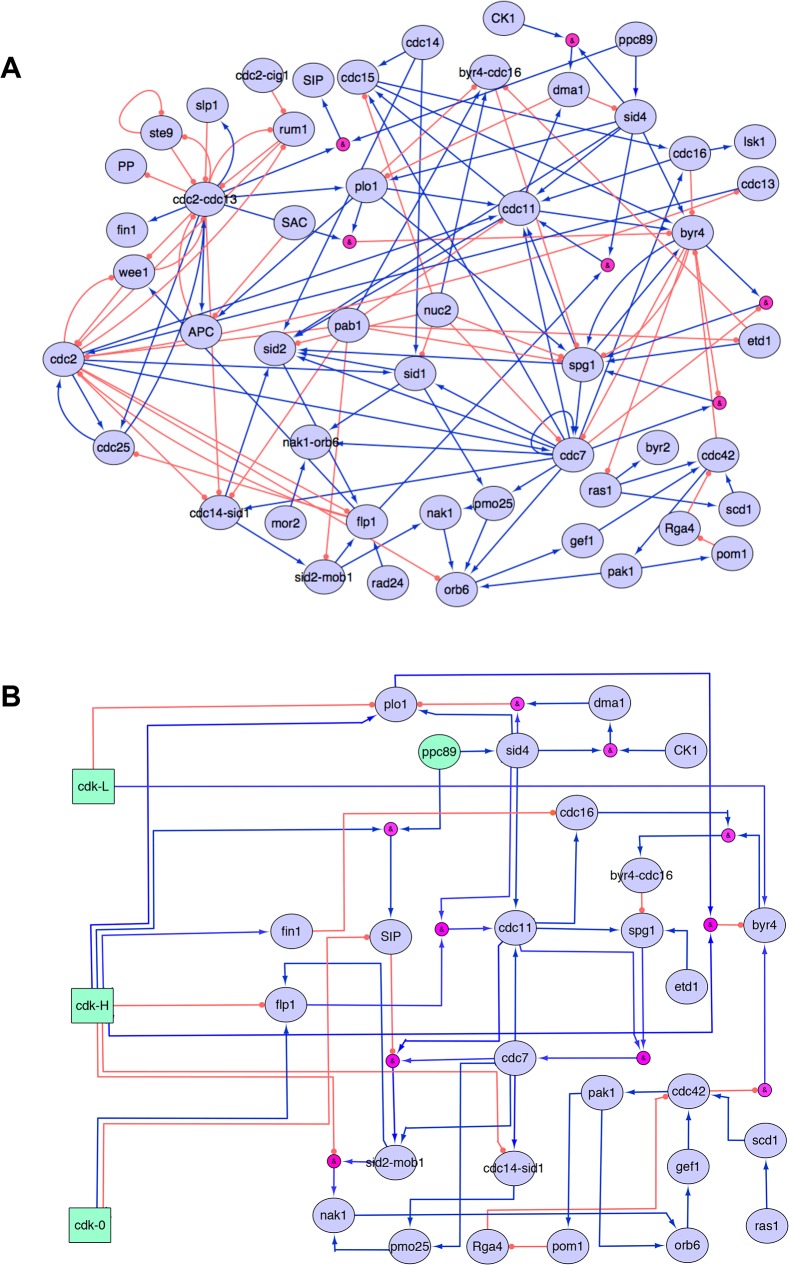
The extended Boolean SIN model. (A) The initial, prior knowledge network, manually re-constructed from the literature. Purple nodes represent proteins and complexes that take part in the regulation of the SIN, and pink nodes represent AND gates. Blue arrows indicate activation events and orange circles inhibition events. Other logical functions, such as AND, OR and NOT regulatory gates are also encoded in the model. SAC: spindle assembly checkpoint, APC: anaphase-promoting complex, PP: protein phosphatase. (B) The final, optimized model, which uses a 3-node representation of CDK activity. Nodes in green are used as switches, and they are turned on to represent different stages of the cell cycle: interphase, early mitosis and late mitosis. Pink nodes represent AND gates. In the case of the Cdc42p regulatory link towards Byr4p, the regulatory rule can be phrased as “NOT Cdc42p activates Byr4p”.

The constructed prior knowledge network (PKN) consists of 50 nodes (gene products, proteins and complexes) and 124 directed edges (Fig 2A). The regulatory information is the result of the curation of 67 published scientific papers (S1 table). The most recently published interaction contained in this model is the inhibitory regulation of CDK and Plo1p upon Byr4p recently published by [46].

The model interactions were classified as activations or inhibitions and they were represented in the network as a combination of Boolean functions that can include AND, OR and NOT [40,47–51].

### Qualitative model simulation

Despite the intensive study of the SIN over the past decades, there is little kinetic data for the protein interactions described in the literature that form the basis for our model. Obtaining such spatiotemporal data is experimentally difficult and represents one of the major challenges in systems biology research. For the simulation of the SIN model we adopted a qualitative Boolean approach, which has been successfully used in several other contexts [30,40,52–62].

In Boolean formalism, each node is characterized by an activation state that can take the values 1 for “active” or 0 for “inactive”, corresponding to the logical values TRUE and FALSE. The activation state can refer to transcription, localization, phosphorylation or other post-translational modifications. For the construction of the SIN model we assumed that for scaffold proteins “TRUE” corresponds to a state that permits the assembly of signaling complexes.

The state of each node depends on the state of the nodes regulating it, that is, the state of all the incoming edges, and the rules that govern their interaction. The state of all the nodes at a given moment defines the network state. The network transitions from state to state are dictated by the underlying Boolean functions, until it reaches a steady state or a cyclic attractor [48]. The possible trajectories in the state space can be represented by the state transition graph [34,63,64].

In Boolean modeling, time is abstract and can be simulated using diverse strategies such as in a continuous manner, with discrete updates or using probabilistic transitions. In the case of discrete time representation, two main updating schemes can be used during model simulation; synchronous and asynchronous update. The former assumes that all biological events in the system have similar timescales, and all functions are updated simultaneously. In the latter, one function is updated at each time step, which can be deterministic (deterministic asynchronous) or randomly selected (stochastic asynchronous) [65,66]. The asynchronous behavior can be controlled by setting additional rules for time delays and priorities [67,68]. Alternatively, all possible transitions can be generated [33,34,67,69].

Asynchronous deterministic updating was chosen for the SIN model simulation in this study, since it assumes non-synchronous regulatory events, which is likely to reflect the *in vivo* situation. However, the challenge in asynchronous update lies in interpreting the simulation trajectories; in stochastic asynchronous simulations, the same initial state can lead to different trajectories in the state space, due to the stochasticity of the updating scheme [34]. The simulation algorithm used was based on *genysis*, a tool for synchronous and asynchronous modeling of gene regulatory networks, based on reduced ordered binary decision diagrams (ROBDDs) [69]. The algorithm identifies all steady states / attractors that can be reached, by efficiently investigating all possible asynchronous state transitions.

### The use of nodes representing CDK levels as input nodes for SIN activity modeling

Cdc2p/CDK1 influences the SIN both positively and negatively. Active Cdc2p inhibits the SIN early in mitosis; its inactivation is required for septum formation and to establish SIN protein asymmetry [3,4,70–72]. Furthermore, Cdc2p and the Byr4p-Cdc16p GAP may cooperate to prevent septation in interphase [73]. However, Cdc2p and Plo1p also collaborate positively to ensure removal of Byr4p from the SPBs and facilitate SIN signaling in anaphase [46]. Failure to increase CDK levels during early mitosis will block cytokinesis, since the cells do not enter mitosis. However, constant, high CDK levels through mitosis will block cytokinesis. Thus, CDK levels need to increase to permit entry into mitosis, after which cytokinesis will occur. However, this will only happen once CDK activity decreases to a very low level, and cells exit mitosis. The model must therefore accommodate these CDK-dependent regulatory events.

Towards this goal, we introduced three independent nodes for CDK, representing the CDK levels before, during and after mitosis. CDK-L corresponds to the low CDK levels during interphase; these prevent re-replication of DNA, but are insufficient for entry to mitosis [74,75], CDK-H represents the high level of CDK activity found in early mitosis. Finally, CDK-0 represents the very low CDK activity in late mitosis as cells undergo the M-G1 transition. This multi-node representation of CDK allows us to describe the SIN-related phenotypes corresponding to several stages of the cell cycle, using the CDK nodes as inputs. For example, setting CDK-L constantly on, indicates that we are simulating the events during interphase, while, CDK-H on represents early mitosis and CDK-0 on represents late mitosis (Fig 2B). It should be stressed that the 3 CDK nodes are not regulated themselves, but are rather used as control (i.e. input) nodes for the system’s simulation. For this reason, there are no incoming regulatory links towards the CDK nodes (CDK-L, CDK-H and CDK-0) (Fig 2B).

### Model refinement and simulation results

This model configuration that uses CDK levels as control nodes for the simulation of cell cycle events, allowed us to clearly define the expected steady states of the system and set our refinement strategy. First, we attributed the PKN interactions involving CDK to the correct CDK node. For example, an activation link from CDK-H was added towards Plo1p (Fig 2B), which in turn will reinforce the activity of CDK in a positive feedback loop [28,76]. Following the attribution step, the model was evaluated using a number of well characterized *in silico* perturbations whose phenotypic consequences are known; knock-out of *cdc11*, *spg1*, *cdc16*, *byr4*, and *cdc7*. For the evaluation, the above 5 knock-out perturbations were simulated, by setting the corresponding node to 0 throughout simulation. A fixed set of nodes, with activation states indicative of the expected phenotype was selected to score the model’s ability to correctly reproduce the mutation outcomes. The scoring set includes *sid4*, *cdc11*, *byr4-cdc16*, *spg1*, *cdc7*, *sid2-mob1* and *sid1-cdc14*. For each *in silico* perturbation, the resulting steady states were evaluated according to the number of the scoring set nodes that had the expected activation state (see S1 Fig for a list of the scoring set expected states).

We proceeded by refining the connections within the network. A refinement cycle consisted of altering an edge of the network, perturbing the model and evaluating the simulation outcome of the perturbations test set. The alterations could involve additions and deletions of regulatory edges, or modifications of the existing regulatory rules. The reasoning behind each change of the model’s regulatory rules was based on several factors, such as the confidence level of each interaction, coupled with information from the published literature, as well as forming alternative logical rules of the given information to better represent the biological reality of the interaction. For example, “A inhibits B” can be alternatively encoded as “NOT A activates B”, and is more suited for cases where the inhibition is not dominant. During this process we maintained the known, required connections of the model and minimized the model’s complexity by removing nodes that no longer served any regulatory role in the model. An example of the latter is the removal of cell cycle regulatory elements such as Cdc25p, Wee1p, Slp1p and Rum1p, to simplify the cell cycle representation by using multi-node CDK. The final, optimized network is presented in Fig 2B. A full list of the edges comprising the final network, together with the justification for the inclusion of each edge, can be found at the supplementary material (S2 Table), as well as the model in genYsis and SBML-Qual format (S1 and S2 Files respectively) [77].

**Fig 3:**
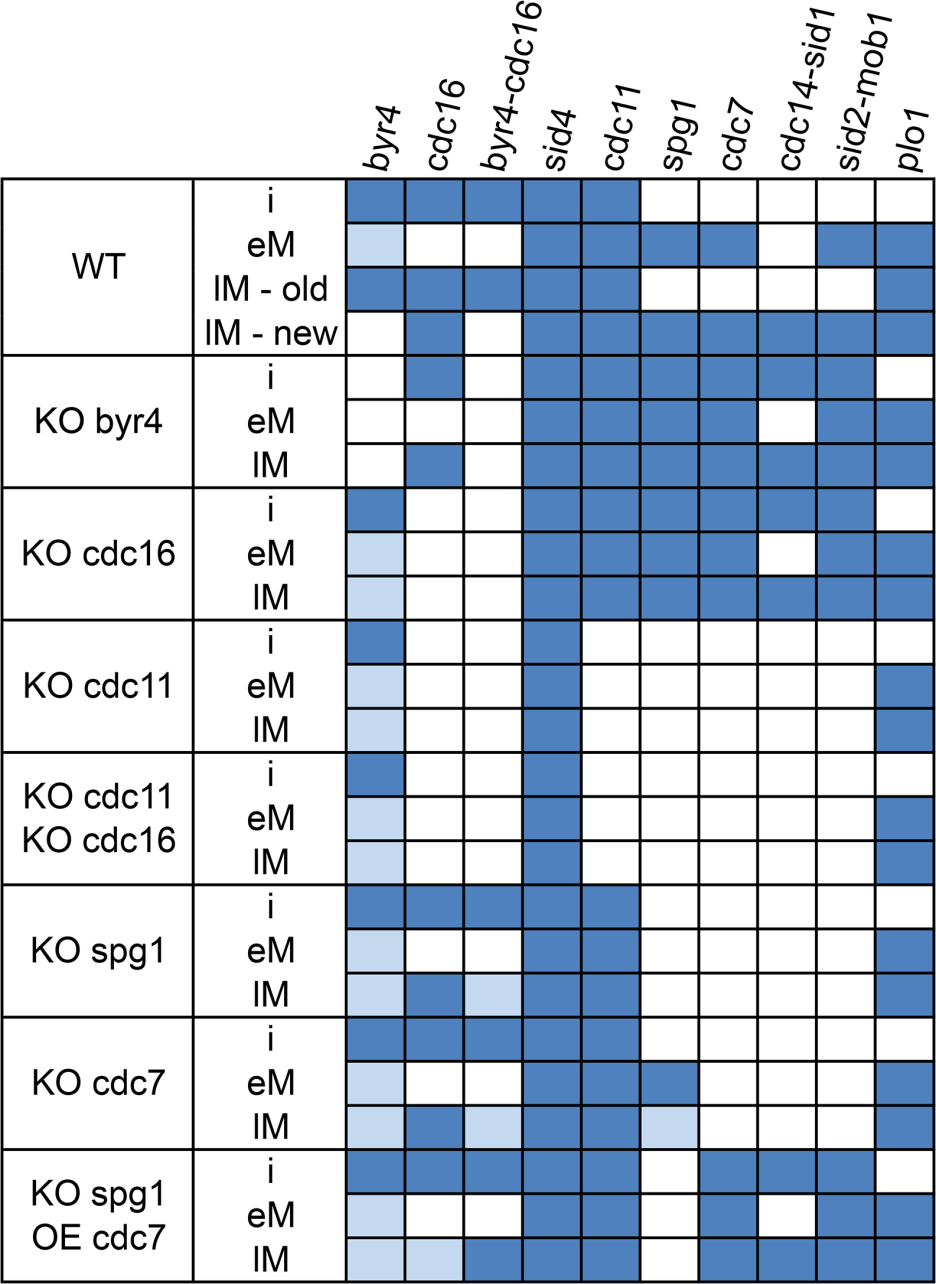
*In silico* steady states of the SIN, in wild type and mutated cells. Steady states deriving from simulations performed on the final model. The boxes on the left indicate the experiments performed, which can be knock-out (KO) or over-expression (OE). When there is more than one gene in the box, it is a double perturbation. For each perturbation, 3 experiments were performed: interphase simulation (indicated as *i*), early mitosis (*eM*) and late mitosis (*lM*, with suffixes *new* and *old* when there are 2 resulting steady states, indicative of late mitosis asymmetry). Blue boxes correspond to active proteins, white to inactive and light blue to proteins that can be either active or inactive at the resulting steady states of the system.

The optimized model was used for *in silico* experiments in which a combination of nodes was perturbed and the phenotypic outcome in the interphase, early mitosis and late mitosis CDK-states were determined. A simulation of the wild type model, where no perturbation is introduced, is presented in Fig 3.

To simulate interphase, CDK-L is set to 1, and Ppc89p is set to 1 as well, to permit “binding” of scaffold proteins to the SPB. In interphase, the model simulation results in a steady state where Byr4p and Cdc16p are present and able to form the GAP complex, therefore active. The scaffold proteins Sid4p and Cdc11p are also present (therefore “active” according to our initial assumption for scaffold molecules), but no SIN signaling occurs due to the inhibitory effect of the Byr4p-Cdc16p GAP.

Early mitosis is simulated by setting CDK-H and Ppc89p to 1. The SIN scaffold is still formed, as expected. Cdc16p is absent from the SPBs in early mitosis, preventing formation of the GAP. This allows SIN signaling to initiate, and we observe that all the main components of the SIN are active (Plo1p, Spg1p, Cdc7p, Sid2p-Mob1p), apart from Cdc14p-Sid1p, which is inhibited by high CDK activity [4,70].

Late mitosis is represented by setting CDK-0 and Ppc89p to 1 during the simulation. There are 2 resulting steady states of the system simulation. In one state, the SIN signaling scaffold is present, the Byr4p-Cdc16p complex is formed, and all SIN components, except Plo1p are inactive. In the other state, Byr4p-Cdc16p is not active, and all proteins of the SIN scaffold and signaling including Cdc14p-Sid1p are active. Intriguingly, these resemble the asymmetric constellation of proteins observed at the old and new SPBs in late anaphase B (see [19,20,29] for review), with the exception of Sid2p-Mob1p, which is present on both SPBs, but only active in one of the two states of the model. Setting GAP function to 0 abolishes the state that resembles the old SPB. Though it is often assumed to be the case, there is scant evidence to support the view that localization of SIN proteins to the SPB is a faithful readout of their *in vivo* activity. There is no data addressing whether Sid2p signals from one or two SPBs in late anaphase. Future experiments will investigate this. A detailed heatmap showing the activation state of all nodes of the model for all experiments presented herein can be found in the supplementary material (S2 Fig).

### Assessing experimentally validated *in silico* perturbations for the model evaluation

The optimized model can describe the SIN related events during interphase, early and late mitosis. In order to evaluate the model’s ability to describe current knowledge regarding *S*. *pombe* mutants, we performed a series of *in silico* knock-out and constant activation experiments mimicking those described in the literature that have an established phenotype. Fig 3 summarizes the steady states yielded after simulating interphase, early and late mitosis behavior of core gene mutants. Interestingly, in all the *in silico* experiments we obtained steady states where the nodes displayed, overall, the expected activation state. More specifically, *cdc11* knock-out completely blocks septation. Both, *byr4* knock-out and *cdc16* knock-out have the same effect, which is failure to inhibit SIN signaling, and therefore SIN triggering in interphase. In a knock-out of either *spg1* or *cdc7*, signaling fails, with Spg1p still getting activated in *cdc7* deletion, indicating that Spg1p acts upstream of Cdc7p, as experimentally proven.

**Fig 4:**
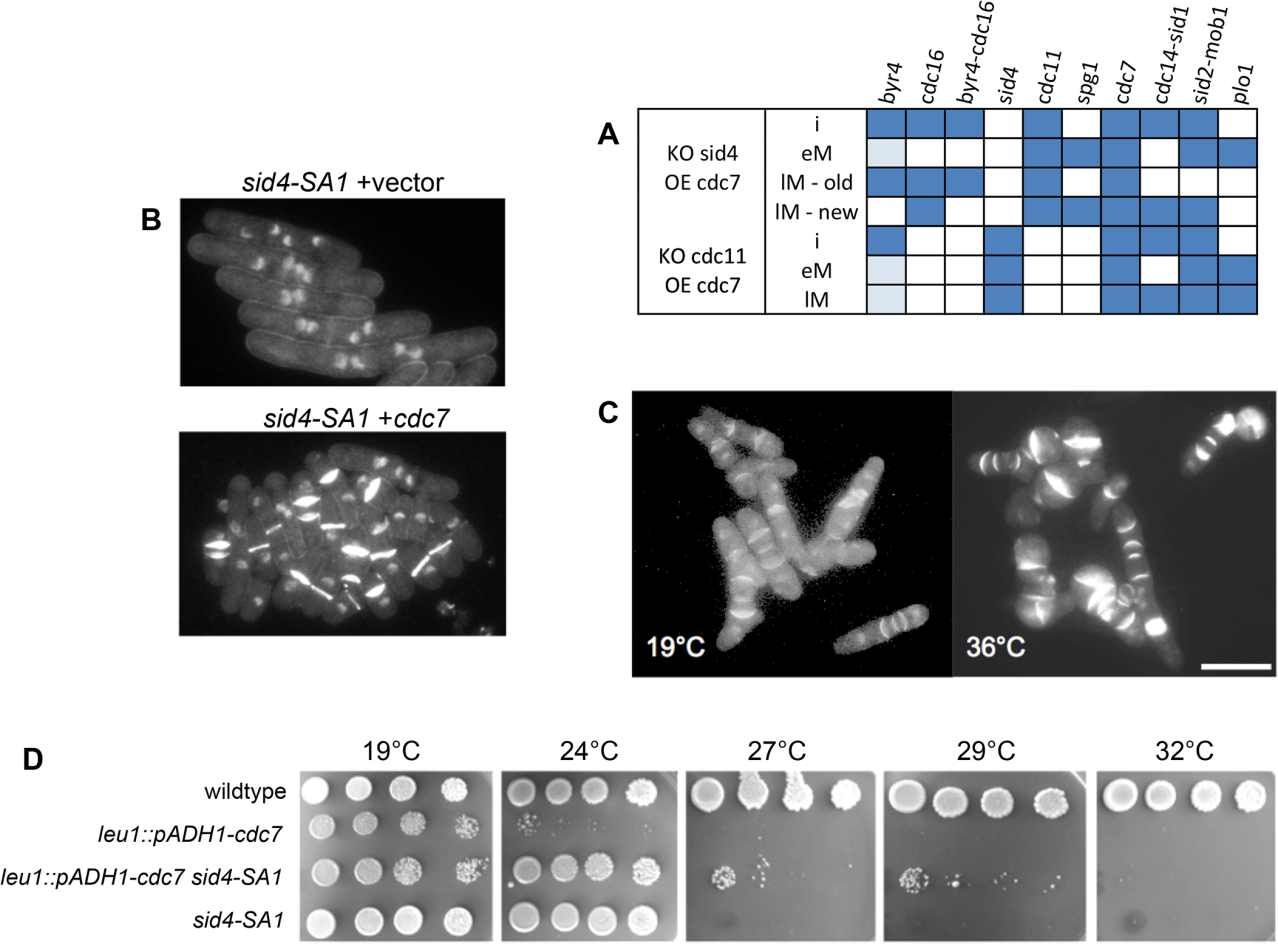
Cdc7p over-expression in a *sid4* mutant will result in septation. (A) Steady states of *in silico*, double mutation experiments. The model predicts that in the absence of SIN scaffold proteins (Cdc11p or Sid4p) and over-expression of Cdc7p, the cell will septate. (B) *sid4-SA1 leu1-32* was transformed with a REP1-based plasmids [80] expressing *cdc7*; empty vector served as a control. Cells were grown to exponential phase in EMM2 medium at 25°C containing 2mM thiamine. Expression was induced by washing with EMM2 and growth for 16h at 25°C; cells were then shifted to 36°C for 5h, fixed, and stained with DAPI and Calcofluor as described [81]. Note that the cells carrying empty vector have become elongated and multinucleated, while 75% of cells expressing *cdc7* have one or more septa. The scale bar represents 10 μm. (C) The strain *leu1*::*pADH1-cdc7* was grown to exponential phase in YE medium at 19°C. A sample was taken and cells were fixed and stained with DAPI and Calcofluor. The remainder of the culture was incubated for 5h at 36°C before fixation. Note the elevated percentage of septated cells. The scale bar represents 10 μm. (D) The indicated strains were grown to exponential phase in YE medium, counted, and diluted to 10^6^ ml^-1^. 10 μl of serial 5-fold dilutions were spotted on plates, allowed to dry and then incubated at the indicated temperature until the wild-type control had formed colonies.

Apart from the experiments that were used as training set for the model refinement, we performed double mutant experiments towards which the model had not been optimized (test set). These experiments assess the predictive value of the model, as the *in silico* predictions are in accordance with the expected results. Specifically, the double deletion of *cdc11* and *cdc16* simulation predicts that cells should not septate, as shown in Fig 3, with supporting evidence from the literature [78]. A Cdc7p over-expression in an *spg1* deletion mutant will septate, in agreement with *in vivo* studies [13]. Moreover, Cdc7p over-expression will produce septation in the absence of Cdc11p (Fig 4A), as confirmed by the literature [79]. In this project, setting a node to 1 throughout the simulation has been used to simulate over-expression *in silico*, except in cases where it is known that the over-expression phenotype results from an indirect effect, such as the titration of another protein.

Other *in silico* experiments performed during the optimization provided us with great insights into potential knowledge gaps regarding SIN regulation, as well as the limitations of our model. One such example was a prediction that a *byr4*-null *sid4*-null should septate. When this was tested *in vivo*, the double mutant cells did not septate. This allowed us to refine the model, by identifying regulatory links that would permit this state to be achieved and target them as candidates for edge deletion. Moreover, the *nuc2* inhibitory links that were present in the PKN revealed our limitation of describing events that occur at the end of septation and the incomplete regulatory inputs to *cdc16* helped us discover a potential link with *fin1*. The *nuc2* and *fin1* cases are discussed in detail below.

### Does Nuc2p have a role in interphase?

Increased expression of the APC/C component *nuc2* blocks septation, while incubation of *nuc2-663* at low restrictive temperature results in cutting of the cell [82,83]. Analysis of how the SIN is reset at the end of mitosis revealed an APC/C-independent role for Nuc2p [84]. Nuc2p interferes with formation of the Cdc7p-Spg1p complex, possibly by stimulating the GAP activity of Byr4p-Cdc16p. Since our current model does not include resetting of the SIN, we tested whether the inhibitory link of *nuc2* towards the Cdc7p-Spg1p complex should be maintained. If it is required, then it might indicate a role for Nuc2p in regulating septation in interphase, once the cell has completed the M-G1 transition. We therefore modeled the effect of inactivating Nuc2p *in silico* upon SIN behavior in interphase. The predicted outcome when including the Nuc2p inhibitory link was two steady states; one with inactive SIN and one with cells that septate in interphase.

To test whether this could be the case *in vivo*, the strain *nuc2-663 atb2-mCherry leu1-32* was arrested in S-phase by growth in medium containing 12mM hydroxyurea (HU). After 5h at 25°C, cells were shifted to 36°C to inactivate Nuc2p, and samples were analyzed at hourly intervals. Before shift to 36°C *nuc2-663* (97%; N = 403 cells) and *nuc2* ^*+*^ cells (97%; N = 480) were mononucleate with no septum; the interphase arrest was confirmed by the presence of an interphase array of microtubules (data not shown). Following shift of the cultures to 36°C, the majority of *nuc2* ^*+*^ cells remained in interphase for three hours, as judged by the continued presence of interphasic microtubules and the absence of a spindle (97%; N = 498, and 90%; N = 400 at, 1 and 2h respectively;). The *nuc2-663* cells maintained the hydroxyurea arrest less efficiently, (89%; N = 319, and 86%; N = 636 at 1, and 2h, respectively). When *nuc2-663* cells entered mitosis, they arrested with a mitotic spindle (not shown). A fraction of both *nuc2* ^*+*^ and *nuc2-663* cells septated in the first two hours, but this did not exceed 5%. In contrast, previous studies from this lab [73] and the Hagan lab [85] showed that activation of the SIN in interphase-arrested cells by incubation of the *cdc16-116* mutant at 36°C produced >50% of type II (mononucleated, septated cells; defined by Minet et al. [7]) within 100 minutes. The levels of septation observed in this experiment are far lower, and, given the similar levels in *nuc2* ^*+*^ and *nuc2-663*, most likely reflect slippage of the hydroxyurea arrest. This leads us to conclude that Nuc2p does not play a major role in preventing septation in interphase, once the M-G1 transition has been completed.

Contrary to traditional studies, where models are constructed from available data and are then used for the experimental design of predictive simulations, our modeling approach is bidirectional: *in vivo* experiments were performed to choose among refinement strategies during model optimization, as well as the model was used to predict experimental outcomes (Fig 1). The case of *nuc2* regulation is an example of the former. Keeping all *nuc2* SIN-related prior knowledge in the multi-node CDK level model required the presence of a dual inhibitory control of the SIN in interphase by both Byr4p-Cdc16p and Nuc2p. *In vivo* experiments were performed to identify the events that can be described by the model and, consequently, guide its refinement strategy. The *in vivo* data argue against a post START role for Nuc2p, in addition to that ascribed to it at the M-G1 transition. Therefore, *nuc2* was removed from the final, model presented here, which does not describe the M-G1 transition in its current form.

### Fin1p over-expression may contribute to inactivation of the GAP for Spg1p at mitotic onset

Fission yeast has a single orthologue of the conserved never-in-mitosis (nimA) kinase, called *fin1* [86]. Fin1p is not essential, but is important for spindle formation and regulates the affinity of Plo1p to the SPB [87]. *Fin1* mutant cells are delayed in the G2-M transition and Fin1p is in part regulated by Sid2p [88]. This link between *fin1* and the SIN prompted us to include *fin1* in the SIN regulatory circuit.

**Fig 5:**
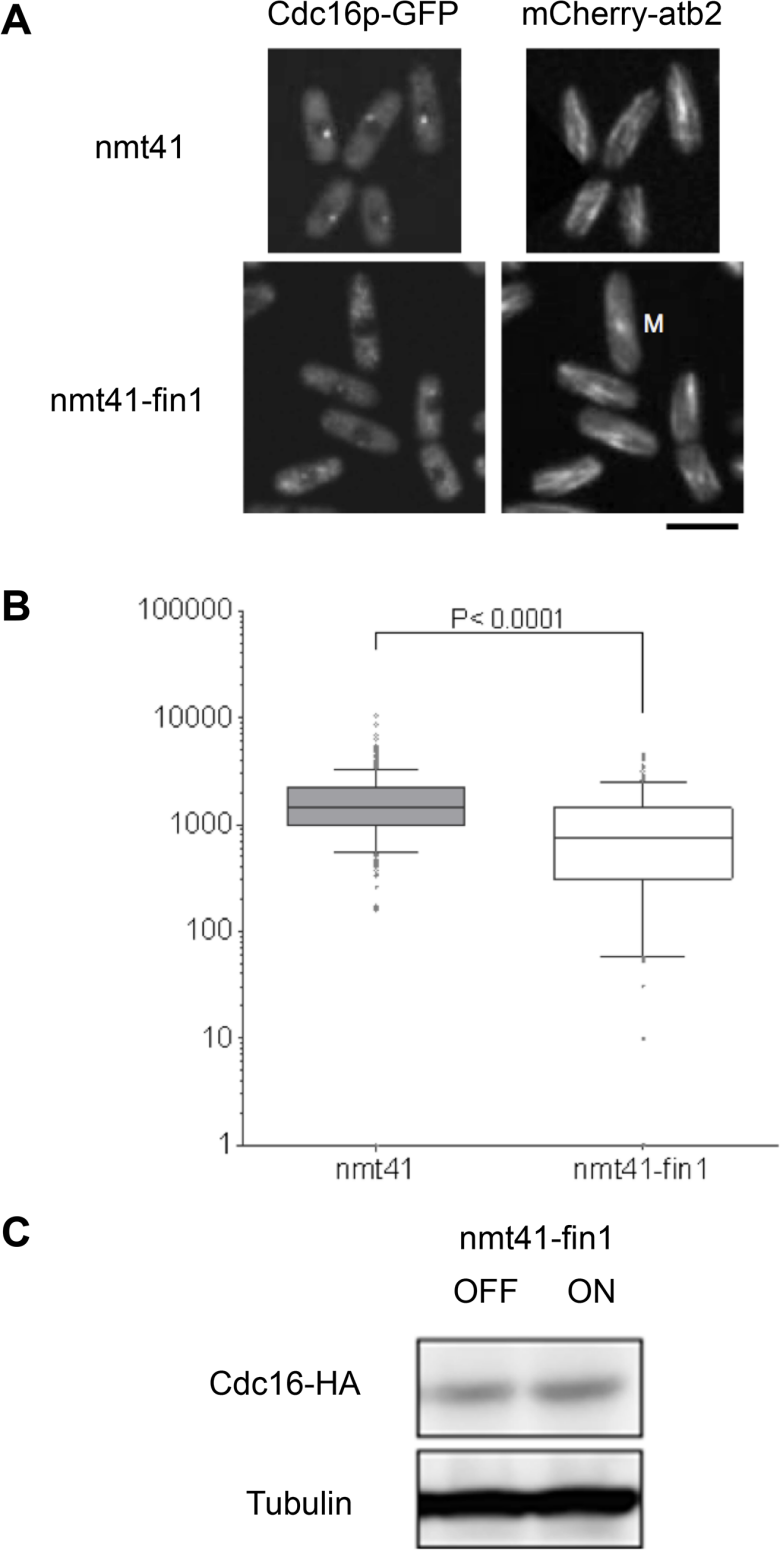
Fin1p over-expression results in Cdc16p disassociation from the SPB. (A) Cells expressing the labeled tubulin marker *leu1*::*m-Cherry-atb2* and *cdc16-GFP* were induced to express *fin1* from the medium strength *nmt41* promoter [80]. Cells transformed with empty vector served as control. Cells were grown in medium without thiamine for 27h at 25°C. Cells were imaged and the intensity of SPB associated cdc16-GFP signal was analyzed as described in [12]. The panel shows *m-Cherry-atb2 leu1*::*cdc16-GFP(ura4* ^*+*^) cells bearing REP41 or REP41-fin1. The scale bar is 10 μm. (B) The SPB associated signal was determined in interphase cells in each strain. Since *REP41-fin1* eventually leads to a mitotic arrest [87] interphase cells were identified by the presence of an interphasic microtubule array. The box shows 25%-75% range for the population, the line indicates the median. The bars indicate 10% and 90% range for the population, and dots indicate more extreme individual values. The y-axis shows fluorescence intensity on an arbitrary scale. (C) Cells bearing the leu1::*cdc16-HA* allele were induced to express *fin1 (ON)* by growing them in defined minimal medium [81] in the presence (OFF) or absence (ON) of 2mM thiamine. Protein extracts were prepared 27h after induction and analyzed by western blotting using monoclonal antibody 12CA5. The anti-α-tubulin monoclonal antibody TAT-1 [90] was used as a control.

In the PKN of the model there were no negative regulators targeting GAP components during early mitosis, which resulted in suboptimal outcomes during the simulations of early mitosis; i.e. the simulation would produce a steady state where the GAP remained active in early mitosis. Since removal of the SIN GAP from the SPB is an early step in the activation of the SIN after entry into mitosis [12,74,89], we modeled whether GAP components could be regulated by *fin1*. Since Cdc16p contains several sites matching the established consensus for mammalian Nek2 (one of the orthologues of nimA), the effect of increased expression of *fin1* on Cdc16p localization was investigated by *in vivo* experiments. Expression of *fin1* from the medium strength nmt-41 promoter [80] resulted in partial displacement of Cdc16p-GFP from SPBs in interphase cells (Fig 5A). Quantification of the SPB-associated signal of Cdc16p-GFP in interphase cells revealed that it was significantly decreased upon expression of *fin1* (Fig 5B). This was not due to a significant alteration of the steady state level of Cdc16p (Fig 5C).

Interestingly, the decreased level of Cdc16p-GFP at the SPB resulted in 24% of REP41-fin1 cells forming one or more septa in interphase, compared to <1% in the empty-vector control. This is consistent with previous studies [73,85], which demonstrated that inactivation of *cdc16-116* in interphase cells promotes septation in interphasic cells. Therefore, the reduced level of Cdc16p-GFP at the SPB may decrease the extent to which the SIN is inhibited by lowering the amount of GAP available to inhibit Spg1p signaling.

Previous studies have shown that in the absence of GAP function, Fin1p acts as an inhibitor of the SIN [85]. This study suggests that increased levels of Fin1p result in the reduction of Cdc16p levels at the SPB, and therefore potentially to the activation of the SIN at the entry into mitosis. This may point to a dual role of Fin1p in SIN regulation, which will be addressed in future studies. Fin1p is implicated both in mitotic commitment, and in SIN regulation [85,88]. Expression of *fin1* promotes recruitment of Plo1p to the SPB in interphase cells [87], and Plo1p is involved in the displacement of Byr4p from the SPB in anaphase [46]. Future studies will examine the mechanism by which Fin1p contributes to the decrease in Cdc16p at the SPB.

### An unexpected prediction: cells overexpressing Cdc7p will septate in the absence of Cdc11p or Sid4p

The final, optimized model describes the existing knowledge of the SIN, in wild type and known mutants. One of the main goals of developing this Boolean model was to use it predictively by performing *in silico* perturbations of interesting and/or experimentally challenging mutants. The regulatory relationships described in this model predict that increased expression of Cdc7p should produce septation in the absence of Cdc11p and Sid4p (Fig 4A). Previous studies have shown that Spg1p overexpression will induce septation and permit colony formation in a *cdc11* mutant [13], but not a *sid4* mutant [91]. Moreover, increased expression of Cdc7p will permit *cdc11* mutants to form colonies [79]. In contrast to the situation with Spg1p overexpression, induction of Cdc7p expression from the very strong nmt1 promoter in *sid4*-SA1 at 36°C did not permit colony formation, but septa were formed in the cells (Fig 4B). To test whether increased expression of Cdc7p would permit growth of a *sid4* mutant, *cdc7* was expressed from the ADH1 promoter, integrated at leu1. The *leu1*::*pADH1-cdc7* strain has a very high septation index at 19°C (>90%) and is barely capable of colony formation at 25°C and above (Fig 4D), with cells dying multiseptated at higher temperatures (Fig 4C). The strain *sid4*-SA1 *leu1*::*pADH1-cdc7* was capable of colony formation at 27°C and 29°C (Fig 4D), where neither parental strain could do so. Previous studies have shown that increased expression of *cdc7* increases the level of kinase activity in immunoprecipitates of Cdc7p [79]. This shows that septation can occur if the function of the scaffold proteins is compromised, provided the expression of Cdc7p is sufficiently elevated. This raises the intriguing possibility that SIN signaling in this case originates in the cytoplasm, bypassing the need for assembly on a SPB-associated scaffold. The nature of the SIN protein signaling complexes present in these cells will be the subject of future studies.

## Discussion

In this paper we use qualitative Boolean modeling to represent and explore the regulatory relationships of genes participating in the Septation Initiation Network of fission yeast. Qualitative modeling is a powerful method for systems with restricted kinetic information and it is computationally efficient, allowing for thousands of *in silico* experiments in a short time, even in networks with hundreds of nodes. Moreover, it can be used predictively, to test combination of mutations that would otherwise be time consuming, expensive and/or experimentally challenging to undertake. The value of such models increases significantly when the model is coupled with *in vivo* experiments. Such experiments can be used to evaluate the regulatory rules, help the optimization procedure and test the predictions of the model (Fig 1).

We report the construction of an extended, Boolean model of the SIN network that uses CDK levels as control nodes to simulate SIN related events in interphase, early mitosis and late mitosis. The prior knowledge network was manually curated, providing a trustworthy initial framework that could then be further optimized (Fig 1). Information reported in literature (and used in network databases) can be conflicting, outdated, incomplete or based on *in vitro* knowledge only. Therefore, expert biocuration provides a significant advantage in order to filter the available information and construct a comprehensive network.

We optimized the model using *in silico* experiments with well-established outcomes based on *in vivo* data, in order to recapitulate the SIN state in different stages of the cell cycle (Fig 1). A challenging aspect of qualitative modeling, and especially of asynchronous update, is to interpret the resulting steady states of the simulations. This is because the simulation might result in a number of steady states that are theoretically possible but never reached *in vivo*. Our approach was to use CDK levels as an initial condition for the simulation, indicating the stage of the cell cycle that the simulation corresponds, to reduce unrealistic simulation outcomes. We further restricted the simulation space by taking as a fact that the scaffold has the potential to be constructed at all times by setting the SIN-SPB linker protein Ppc89p to 1.

The optimization process under the controlled environment of CDK switches provided important insights into SIN regulation during the cell cycle. In the case of the *fin1*, the incorrect simulation results that were obtained in early mitosis helped us locate a potential missing link in the PKN. Increased expression of *fin1* removes Cdc16p from the SPB. At present we do not know whether this is by direct phosphorylation of Cdc16p or an indirect effect; this will be the subject of future analysis. However, the important point in this context is that the modeling revealed the requirement for an additional control point to turn off the GAP in early mitosis. The optimization strategy was also useful in evaluating the limitations of our model. An example of this is the role of Nuc2p in SIN regulation. In the PKN there were several inhibitory links from Nuc2p to SIN kinases, indicating the events in SIN resetting, after septation [84]. The use of CDK switches restricts the cell cycle events that can be modeled, and our model does not presently incorporate resetting of the SIN at the M-G1 transition. Our modeling predicted that if Nuc2p continued to activate the GAP in interphase, extending the role proposed for it at the M-G1 transition [84], then its inactivation in post-START cells could result in septum formation; *in vivo* analysis showed this was not the case. Thus, the modeling was useful in this case to define the possible limits of the extent of the time-window in which Nuc2p is active towards the SIN.

The great value of creating an optimized qualitative model is that it can then be used predictively to perform difficult or iconoclastic experiments *in silico*. We focused on testing whether an over-expression of SIN kinases would rescue SIN scaffold mutants. The model’s prediction was that over-expression of Cdc7p in a *cdc11* or *sid4* knock-out will still septate, a prediction that was experimentally validated. The model can be used in the future for any combination of gene mutants, and hopefully provide interesting hypotheses that can be tested experimentally. Future studies will aim to model the M-G1 transition, and to incorporate spatial components into the model (protein localization to one or both SPB, cytoplasm or division site). This will be facilitated by the incorporation of the cell cycle Boolean module of fission yeast, by Davidich & Bornholdt [30]. We will also incorporate multivariate nodes to simulate the effect of changes in the post-translational modifications of SIN proteins during the cell cycle [41,92,93]. This should allow modeling of the role of the asymmetry of SPBs with regard to SIN protein association, building upon the analysis performed by Bajpai et al. [41]. Future versions of the model will attempt to incorporate Etd1p. Though its effects upon SIN signaling are clear, the published analyses do not provide a sufficiently clear, direct link to SIN components to permit its unequivocal incorporation into the model presented here.

Our extended, Boolean model of the SIN can be used by the scientific community for testing various hypotheses *in silico*, including multiple gene perturbations that can be experimentally challenging. The model can be reduced to a minimum number of nodes and still capture the system steady states (see S3 File). Though a reductive approach can be a useful aid in understanding the information flow in the system, the greater complexity of the extended model system increases the predictive value of the model, as we can use the model nodes for testing the desired experimental scenarios.

Finally, it is worth noting that qualitative models such as the one presented here are oversimplifications of the actual regulatory processes; in our case of the regulation of the SIN. With advances in live monitoring of cell division and development of new fluorescent probes, we should be able to generate more accurate quantitative models for such a system. Our approach is nevertheless an important step towards a more comprehensive model that recapitulates known biology of the SIN and can be used as a hypothesis generator for complex experimental design.

## Materials and Methods

### Literature database construction

For the construction of the PKN, several online resources were curated to retrieve SIN relevant information, such as Pathguide, Pubmed, iHOP, iRefWeb, Scholar Google, PriME and UniProtKB/Swiss-Prot. The collected information was stored in a structure formed of pairwise interactions and regulations that includes information about participating components, the origin of publications (PMID) where the interaction was mentioned and a confidence level as an evidence tag from the biocurator. In detail, the database contains the following columns:

1. Node 1: The name of the first element of the interaction, the one that acts as activator or inhibitor
2. Action: A symbol characterizing the type of interaction as activation (->) or inhibition (-|)
3. Node 2: The name of the second element of the interaction, the one that gets activated or inhibited
4. Node 1 type: The type of node 1. Can be protein, complex or miRNA
5. Node 2 type: The type of node 2. Can be protein or miRNA
6. UniProt ID 1: Reference of node 1
7. UniProt ID 2: Reference of node 2
8. PMID: Literature reference of the interaction
9. Class: A letter characterizing the confidence level of the interaction. It can be one of the following:
*Sure (S)*, when the interaction is confirmed or known in textbook, and/or already in the UniProt general annotation lines. Sure interactions are generally associated with many PMIDs.
*Unsure (U)*, when the interaction is shown once and/or not confirmed by others, or when the authors are not confident about the results.
*Inferred (I)*, when there are no results for the network in question, but the interaction was found in different cell types and/or organisms. It may also refer to cases where the information is inferred to all protein isoforms of a gene without confirmed results. Inferred interactions might be associated with more than one PMIDs.
*Contradictory (C)*, when the interaction is based on contradictory results.
10. Evidence tag: Short extract from the publication where the interaction is mentioned.

### GenYsis Boolean modeling toolbox

All Boolean simulations of the SIN model, including the identification of wild type attractors and *in silico* perturbation experiments, we performed using the *genYsis* Boolean modeling toolbox. *GenYsis* uses reduced ordered binary decision diagrams (ROBDDs) in order to efficiently compute attractors and steady states of large networks. ROBDDs are directed acyclic graphs that can represent Boolean functions efficiently, and are computationally suitable for complex Boolean operations. To map gene regulatory networks on ROBDDs the network has to be transformed into Boolean functions that represent the dynamics of the model. All the operations that can be performed on Boolean functions can also be performed on their corresponding ROBDD representations [69]. The simulation modes available with genYsis include synchronous and asynchronous updating. In both cases, the user has the possibility of performing *in silico* perturbations by fixing the activation state of one or multiple components during simulation. The perturbation possibilities comprise (a) knock-out experiments, were the selected components are set to be inactive during the whole simulation, (b) over-expression experiments, were the selected components are set to be active during the whole simulation, and (c) initial state experiments, where the selected components have a fixed activation state at the beginning of the simulation that is thereafter allowed to change according to the regulatory rules. The software binaries of *genYsis* are available for Linux (64 bits gcc version 4.4.5, Debian 4.4.5–8) and Mac OS X (64 bits gcc version 4.2.1, Mac OS X 10.8.5) at http://www.vital-it.ch/software/genYsis.

### Fission yeast techniques

#### (A) Media

Growth and manipulation of *S*. *pombe* was performed according to standard protocols [81]. Defined medium was EMM2 with supplements at 100 mg/l as required, and complete medium was YE [81]. Cell number was determined using a hemocytometer. For the induction of *nmt1* regulated genes, cells were grown to exponential phase (approx. 2-3x 10^6^ ml^-1^) in EMM2 containing 2 μM thiamine with additional supplements as required. Cells were washed twice in medium without thiamine, and then grown for the time indicated in the Fig legends.

#### (B) Molecular and Genetic analyses

Strains were constructed by standard genetic methods. Vectors [80,94] and *cdc7* plasmids [79] have been described previously.

#### (C) Imaging and image analysis

Living cells were imaged using a U-Plan-S-Apo 60× N.A. 1.42 objective lens mounted on an Olympus IX-81 spinning disc confocal microscope. The temperature was maintained using a custom-built heating system. Fixed cells were photographed on a Zeiss Axiophot microscope using a Zeiss 100x NA 1.4 PLAN-apochromat lens. Images were captured on a Nikon Coolpix camera. Level adjustment and cropping were performed using Adobe Photoshop CS6.

DAPI and Calcofluor staining was performed on cells that had been harvested by centrifugation, washed, and fixed with cold 70% (v/v) ethanol, as described previously [95]. Microscopy analysis of living cells was performed as described in [12], using the RodcellJ imageJ plugin [96]. Data were plotted using GraphPad Prism v6. In the whisker plots the box shows 25%-75% range for the population, the line indicates the median. The bars indicate 10% and 90% range for the population, and dots indicate more extreme individual values.

## Supporting Information

S1 File The SIN model in genYsis format. The final model in genYsis format can be used for any combination of *in silico* experiments using the genYsis software.
(ZIP)

S2 File The SIN model in SMBL qual format. The final model in SBML qual format can be used to perform additional analyses in most qualitative modeling platforms.
(ZIP)

S3 File Model reduction analysis. A reduction analysis performed using GINsim [97], highlighting the information flow that is necessary for the maintenance of the system’s steady states.
(PDF)

S1 Fig The model optimization scoring set. The experiments used to score the model candidates during the optimization phase are represented in the y axis and the proteins used for scoring in the x axis. The table uses the same color coding as the article figures: blue for Boolean state 1, white for Boolean state 0 and light blue for oscillation or, in this case, two alternative steady states with different activation states of the given protein.
(TIF)

S2 Fig Detailed *in silico* results of final model simulations. A detailed heatmap showing the activation state of all nodes of the final model for all experiments presented in this paper.
(TIF)

S1 Table Prior Knowledge Network interaction table. A complete list of the interactions included in the Prior Knowledge Network of the SIN.
(PDF)

S2 Table Final model interaction list. **The** list of interactions comprising the final, optimized model, together on comments justifying their alteration / addition.
(PDF)
