# Supplementary material for: An Extended, Boolean Model of the Septation Initiation Network in S.Pombe Provides Insights into Its Regulation
Source: PLoS One. 2015 Aug 5;10(8):e0134214. doi: 10.1371/journal.pone.0134214 (PMC4526654; doi:10.1371/journal.pone.0134214)
Supplement: S3 File — A reduction analysis performed using GINsim [97], highlighting the information flow that is necessary for the maintenance of the system’s steady states. (PDF) [file pone.0134214.s003.pdf]

Model Reduction Analysis

A useful analysis, when exploring the structure and dynamics of a model, is to reduce it while maintaining its principal dynamical properties. In the case of the SIN Boolean model, this would imply reducing the model while maintaining the attractors resulting from the interphase, early mitosis and late mitosis experiments. Using the model reduction feature of GINsim, a logical modeling toolbox [1], the SIN model was reduced to contain only the basic SIN regulators used for the scoring, as well as the CDK control nodes (Fig. A). The reduced SIN model can be used to capture the information flow of the system. We observe that the CDK-L and CDK-H nodes remain essential for maintaining the system steady states, whereas the absence of CDK (CDK-0 node) is the natural output of the simulation when no breakpoints are active.

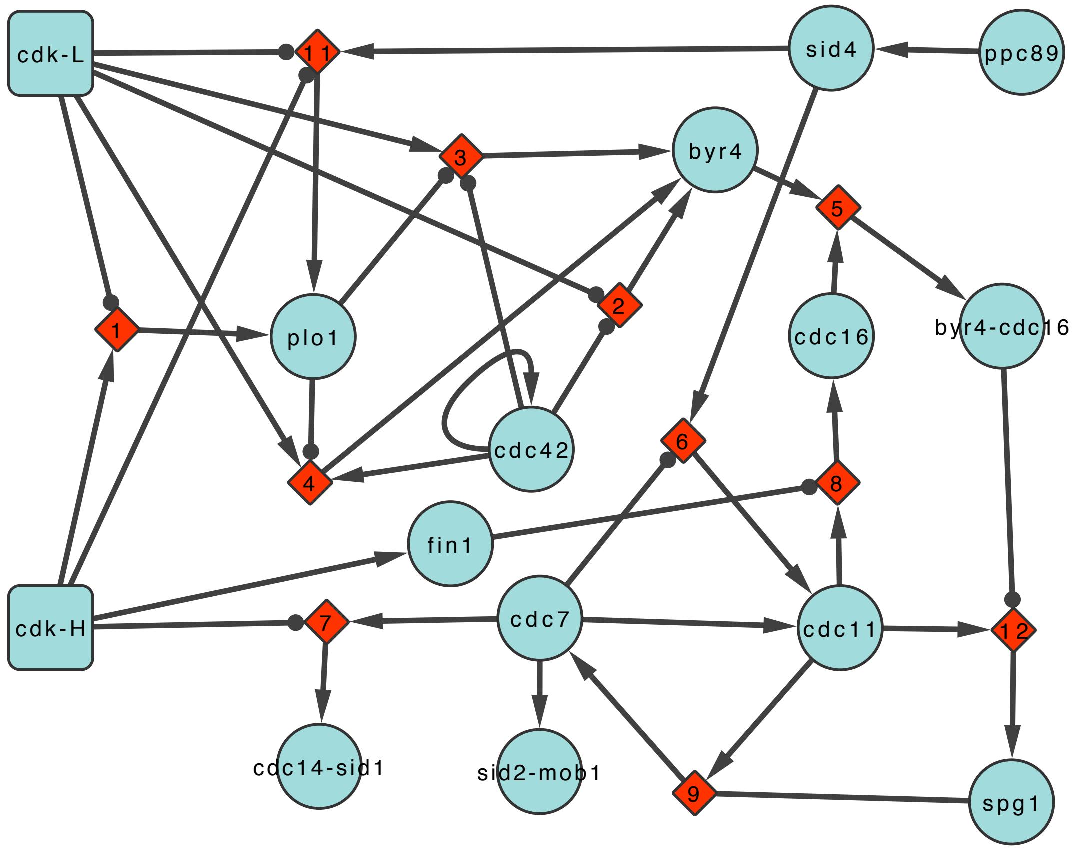

Fig A. A reduced version of the Boolean SIN model.

Network structure after minimizing the SIN model participating components to the main regulators used in the optimization scoring as well as the CDK control nodes.

1. Chaouiya C, Naldi A, Thieffry D (2012) Logical modelling of gene regulatory networks with GINsim. Methods Mol Biol 804: 463-479.
