# Supplementary figures and images for: An Extended, Boolean Model of the Septation Initiation Network in S.Pombe Provides Insights into Its Regulation
Source: PLoS One. 2015 Aug 5;10(8):e0134214. doi: 10.1371/journal.pone.0134214 (PMC4526654; doi:10.1371/journal.pone.0134214)

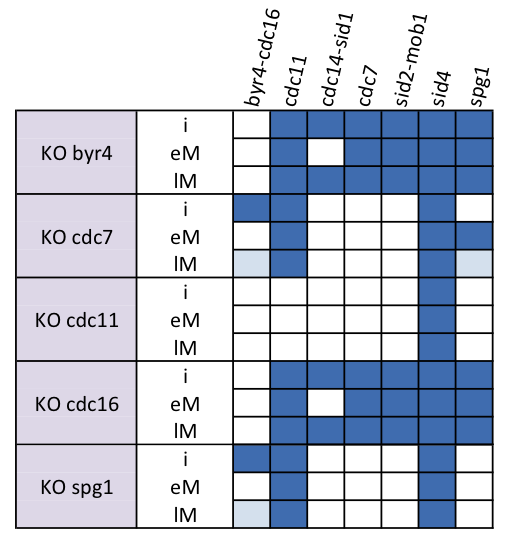

Supplement: S1 Fig — The experiments used to score the model candidates during the optimization phase are represented in the y axis and the proteins used for scoring in the x axis. The table uses the same color coding as the article figures: blue for Boolean state 1, white for Boolean state 0 and light blue for oscillation or, in this case, two alternative steady states with different activation states of the given protein. (TIF) [file pone.0134214.s004.tif]

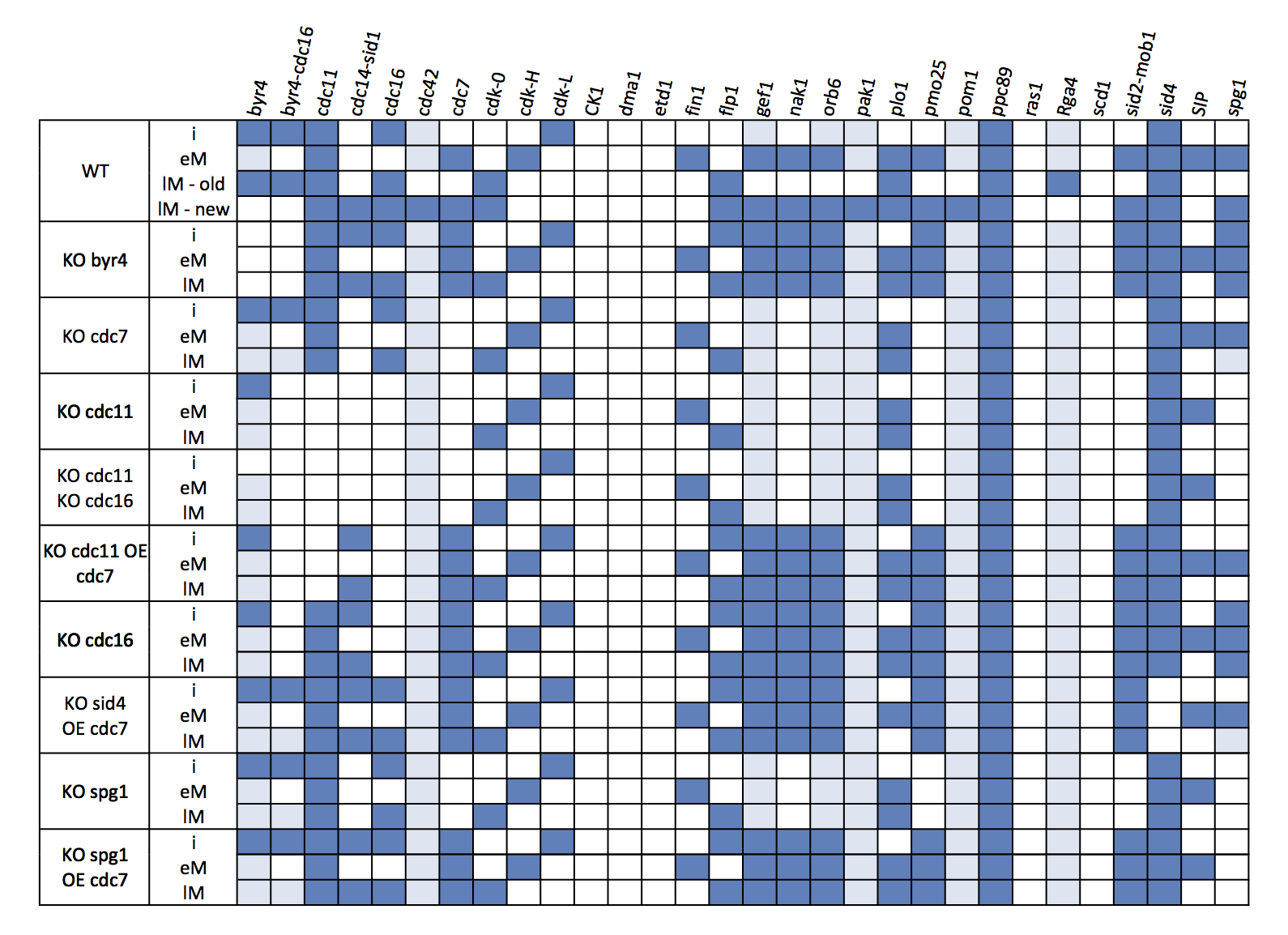

Supplement: S2 Fig — A detailed heatmap showing the activation state of all nodes of the final model for all experiments presented in this paper. (TIF) [file pone.0134214.s005.tif]
