# Supplementary material for: An Extended, Boolean Model of the Septation Initiation Network in S.Pombe Provides Insights into Its Regulation
Source: PLoS One. 2015 Aug 5;10(8):e0134214. doi: 10.1371/journal.pone.0134214 (PMC4526654; doi:10.1371/journal.pone.0134214)
Supplement: S1 Table — A complete list of the interactions included in the Prior Knowledge Network of the SIN. (PDF) [file pone.0134214.s006.pdf]

Node 1	Action	Node 2	Node1 type	Node2 type	UniProtID	UniProtID	PMID (;)	Class	Evidence Tag
byr4-cdc16	-|	spg1	complex	protein	Q10951-P36618	P87027	*10381387;17340144;19305416;9742395;20876564;16691419;18252797;19736319*	S	Cdc16 and Byr4 form a two component GTPase-activating protein (GAP) for Spg1 that inhibits its activity
spg1	->	cdc7	protein	protein	P87027	P41892	19736319;*17340144*;*9742395*;*9203579*;*11514436*;*18252797*;*16691419*;*10381387*;*9420333*;	S	GTP-bound Spg1 recruits its downstream effector, the Cdc7 kinase
spg1	->	byr4	protein	protein	P87027	Q10951	*18252797*	I	Byr4p levels are very low in the absence of Spg1p. These results demonstrate that the steady-state level of Byr4p reflects that of Spg1p.
cdc11	->	byr4	protein	protein	O74473	Q10951	*18252797*	I	Furthermore, after inactivation of SIN scaffold components such as Cdc11p (two alleles) or Sid4p, the slower-migrating form of Byr4p was absent, suggesting that complete phosphorylation of Byr4p requires SPB localisation.
sid4	->	byr4	protein	protein	O60187	Q10951	*18252797*	I	Furthermore, after inactivation of SIN scaffold components such as Cdc11p (two alleles) or Sid4p, the slower-migrating form of Byr4p was absent, suggesting that complete phosphorylation of Byr4p requires SPB localisation.
cdc16	-|	byr4	protein	protein	P36618	Q10951	*18252797*	I	these data are consistent with the view that degradation of Byr4p requires its interaction with Cdc16p
cdc7	->	cdc15	protein	protein	P41892	Q09822	*1527180*	U	we propose that the cdc15 gene encodes a function that is necessary for septum formation and is dependent upon the action of the cdc7, cdc11 and cdc14 products for activation.
cdc11	->	cdc15	protein	protein	O74473	Q09822	*1527180*	U	we propose that the cdc15 gene encodes a function that is necessary for septum formation and is dependent upon the action of the cdc7, cdc11 and cdc14 products for activation.
cdc14	->	cdc15	protein	protein	P36589	Q09822	*1527180*	U	we propose that the cdc15 gene encodes a function that is necessary for septum formation and is dependent upon the action of the cdc7, cdc11 and cdc14 products for activation.
cdc15	->	cdc16	protein	protein	Q09822	P36618	*1527180*	U	Examination of the phenotypes of double mutants of cdc15 with the other four genes suggests that cdc15 functions downstream from cdc7, cdc11 and cdc14, yet before cdc16.
wee1	-|	cdc2	protein	protein	P07527	P04551	*1825699*;*15917811*;*20805322*	S	Wee1 phosphorylates and inhibits cdc2
nak1	->	orb6	protein	protein	O75011	O13310	20805322;*16096637*	S	Orb6 kinase is activated by the GC kinase Nak1;The exact mechanism by which Nak1 activates Orb6 remains unclear.
cdc7	->	orb6	protein	protein	P41892	O13310	*20805322*	S	Cdc7 was required for the large peak in the Orb6 activity after completion of mitosis
pmo25	->	nak1	protein	protein	Q9P7Q8	O75011	20805322;*16096637;*	S	the known dependence of Nak1 activity on Pmo25
mor2	->	nak1-orb6	protein	complex	Q9HDV6	O75011-O13310	*20805322*	I	the fusion, but not the Nak1 and Orb6 proteins individually or in combination, completely rescued a mutation in the scaffold protein Mor2, suggesting that the key function of Mor2 is to bring Nak1 and Orb6 together
cdc2	-|	orb6	protein	protein	P04551	O13310	*20805322*	I	The initial drop in Orb6 activity may be triggered directly or indirectly by Cdk1 activation and mitotic entry, and then when Cdk1 activity drops in anaphase, the SIN becomes active and<br>maintains inhibition of the MOR pathway until cytokinesis is complete.
etd1	->	spg1	protein	protein	Q9UTR4	P87027	*19736319*	S	Our results suggest that the key function of Etd1 is to cause the hyperactivation of Spg1 that occurs in late anaphase.
cdc7	->	sid2	protein	protein	P41892	Q09898	19736319;10459013;10775265	S	SPB-localized Cdc7 then promotes activation of the Sid2 protein kinase
cdc11	->	sid2	protein	protein	O74473	Q09898	*10459013*;*15062098*	I	Cdc11p(488–660) was the smallest tested Cdc11p region that showed a positive interaction with Sid2p. Using a similar strategy, we found that the N terminus but not the kinase domain of Sid2p directed its interaction with Cdc11p
cdc14	->	sid2	protein	protein	P36589	Q09898	*10459013*	I	Both Sid2p localization to the division site and activity depend on the function of all of the other septation initiation genes: cdc7, cdc11, cdc14, sid1, spg1, and sid4.
sid1	->	sid2	protein	protein	O14305	Q09898	*10459013*;*10775265*	S	Both Sid2p localization to the division site and activity depend on the function of all of the other septation initiation genes: cdc7, cdc11, cdc14, sid1, spg1, and sid4; […] consistent with Sid1p functioning upstream of Sid2p
spg1	->	sid2	protein	protein	P87027	Q09898	*10459013*	S	Both Sid2p localization to the division site and activity depend on the function of all of the other septation initiation genes: cdc7, cdc11, cdc14, sid1, spg1, and sid4.
sid4	->	sid2	protein	protein	O60187	Q09898	*10459013*	U	Both Sid2p localization to the division site and activity depend on the function of all of the other septation initiation genes: cdc7, cdc11, cdc14, sid1, spg1, and sid4.
cdc13	->	cdc2	protein	protein	P10815	P04551	19736319;*16390871*	S	Inactivation of Cdc2 appears to result from the inability to form complexes with its mitotic cyclin partner Cdc13; Anaphase SIN activation appears to be governed at least in part by loss of Cdk1 activity, which occurs when cyclin B is degraded in anaphase

cdc2	-|	cdc13	protein	protein	P04551	P10815	8106468	I	Degradation of cyclin B is triggered by increased levels of active cdc2 and is required for exit from mitosis.
ras1	->	byr2	protein	protein	P08647	P28829	8682866;16931912	S	ras1 binds to and presumably activates the byr2 kinase
ras1	->	cdc42	protein	protein	P08647	Q01112	8682866;20876564	S	ras1-scd1-cdc42 pathway
ras1	->	scd1	protein	protein	P08647	P40995	8682866;11063680	U	ras1 binds and presumably activates the scd1 protein
byr4	-|	ras1	protein	protein	Q10951	P08647	8682866	U	A novel suppressor of ras1 in fission yeast, byr4, is a dosage-dependent inhibitor of cytokinesis
cdc15	->	byr4	protein	protein	Q09822	Q10951	8682866	U	The effect of the cdc15-136 mutation on the byr4 protein also suggests a possible interaction between cdc15 and byr4.
cdc2	->	cdc25	protein	protein	P04551	P06652	14765109	S	At mitotic commitment, Cdk1p participates in its own regulation by activating the mitotic inducing phosphatase, Cdc25p, and inhibiting the opposing kinase, Wee1p. (And cf Paul Nurse, UNIL 16 Sept 2011)
cdc2	-|	wee1	protein	protein	P04551	P07527	14765109	S	At mitotic commitment, Cdk1p participates in its own regulation by activating the mitotic inducing phosphatase, Cdc25p, and inhibiting the opposing kinase, Wee1p. (And cf Paul Nurse, UNIL 16 Sept 2011)
flp1	->	wee1	protein	protein	Q9P7H1	P07527	14765109	U	Clp1p might similarly dephosphorylate and activateWee1p at mitotic exit and/or promote its stabilization, as Wee1p is known to be destabilized upon mitotic entry in S. pombe (Aligue et al, 1997). In the future, it will be interesting to determine whether Clp1p targets Wee1p
flp1	-|	cdc25	protein	protein	Q9P7H1	P06652	14765109;15911625;15128870	S	Clp1p dephosphorylates and inactivates Cdc25p in late mitosis.
cdc2	-|	flp1	protein	protein	P04551	Q9P7H1	14765109	U	
flp1	-|	cdc2	protein	protein	Q9P7H1	P04551	14765109;19305416	S	our findings suggest that Clp1p contributes to Cdk1p inhibition at least in part by dephosphorylating, destabilizing, and inactivating Cdc25p, thereby disrupting the Cdk1p positive feedback loop.
cdc25	->	cdc2	protein	protein	P06652	P04551	15911625;15107615;18257517	S	Flp1 antagonizes mitotic CDK activity by promoting the phosphorylation of the conserved tyrosine residue Tyr15 in Cdc2, in part by downregulating the Cdc25 phosphatase, which dephosphorylates Tyr15 (And cf Paul Nurse, UNIL 16 Sept 2011)
byr4	-|	cdc7	protein	protein	Q10951	P41892	10799520	S	Byr4 prevents Cdc7 localization to SPBs and septation in interphase cells and is required to asymmetrically localize Cdc7 to mitotic SPBs.
byr4	-|	spg1	protein	protein	Q10951	P87027	10799520	C	These results suggest that Byr4 localization to SPBs maintains Spg1 in an inactive form
pab1	-|	spg1	trimer	protein	Q12702	P87027	20876564	U	A molecular function for PP2A-Pab1 in negative regulation of Spg1 (…)We cannot exclude that PP2A-Pab1 could directly inhibit Spg1; however, we failed to detect physical interactions between Pab1 and this GTPase
pab1	-|	etd1	trimer	protein	Q12702	Q9UTR4	20876564	U	PP2A-Pab1 might function as a negative regulator of Spg1, antagonizing Etd1 activity.
pab1	->	byr4-cdc16	trimer	complex	Q12702	Q10951-P36618	20876564	U	we hypothesize that PP2A-Pab1 inhibition of Spg1 is more likely to operate indirectly, perhaps through activating dephosphorylation of the Cdc16-Byr4 two-component GAP.
etd1	-|	byr4-cdc16	protein	complex	Q9UTR4	Q10951-P36618	20876564	U	Etd1 might antagonize PP2A-Pab1 function through Spg1 GAP(s) inactivation and delocalization rather than by GEF activity stimulating Spg1 nucleotide exchange.
pab1	-|	cdc14-sid1	trimer	complex	Q12702	P36589-O14305	20876564	U	Thus, the rescue of cdc11-119, mob1-R4, and sid1-239 mutants could also result from PP2A-Pab1 being a negative regulator of the Sid1-Cdc14 and Sid2-Mob1 complexes and/or of their assembly at the Cdc11-Sid4 scaffold.
pab1	-|	sid2-mob1	trimer	complex	Q12702	Q09898-O94360	20876564	U	Thus, the rescue of cdc11-119, mob1-R4, and sid1-239 mutants could also result from PP2A-Pab1 being a negative regulator of the Sid1-Cdc14 and Sid2-Mob1 complexes and/or of their assembly at the Cdc11-Sid4 scaffold.
pab1	-|	cdc11	trimer	protein	Q12702	O74473	20876564;12546793;18481970;11707284	S	Protein phosphatase 2A (PP2A) is implicated in dephosphorylation of cdc11p at the end of mitosis
cdc7	->	cdc11	protein	protein	P41892	O74473	8039497;1527180;12546793	S	We demonstrate that mitotic hyperphosphorylation of cdc11p requires the activity of cdc7p
cdc2	->	cdc11	protein	protein	P04551	O74473	11676915	US	cdc2p could be involved in the phosphorylation of cdc11p at the onset of mitosis, given the presence of multiple cdc2p consensus sites in the N-terminal half of the protein and the timing of its activation.
pab1	-|	cdc2	trimer	protein	Q12702	P04551	20876564;11531413;11380623	S	This result suggests that PP2A-Pab1 phosphatase is involved in morphogenesis by inactivating Cdc2 and/or by counteracting its activity through dephosphorylation of Cdc2-phosphorylated substrates
pmo25	->	orb6	protein	protein	Q9P7Q8	O13310	20876564	S	Pmo25 is essential for Orb6 kinase activity

cdc7	->	pmo25	protein	protein	P41892	Q9P7Q8	20876564	I	The localization of Pmo25 at the SPBs and the kinase activities of Nak1-Orb6 during interphase are under the control of the Cdc7 and Sid1 SIN kinases, suggesting a functional linkage between SIN and the network for cell morphogenesis
cdc7	->	nak1-orb6	protein	complex	P41892	O75011-O13310	20876564	I	The localization of Pmo25 at the SPBs and the kinase activities of Nak1-Orb6 during interphase are under the control of the Cdc7 and Sid1 SIN kinases, suggesting a functional linkage between SIN and the network for cell morphogenesis
sid1	->	pmo25	protein	protein	O14305	Q9P7Q8	20876564	I	The localization of Pmo25 at the SPBs and the kinase activities of Nak1-Orb6 during interphase are under the control of the Cdc7 and Sid1 SIN kinases, suggesting a functional linkage between SIN and the network for cell morphogenesis
sid1	->	nak1-orb6	protein	complex	O14305	O75011-O13310	20876564	I	The localization of Pmo25 at the SPBs and the kinase activities of Nak1-Orb6 during interphase are under the control of the Cdc7 and Sid1 SIN kinases, suggesting a functional linkage between SIN and the network for cell morphogenesis
cdc7	->	cdc7	protein	protein	P41892	P41892	16469735	I	we found that Cdc7 self-associates, adding another layer of complexity to SIN organization
byr4	->	spg1	protein	protein	Q10951	P87027	9742395	C	Our results also show that Byr4 binds to Spg1 and that this binding inhibits GTP hydrolysis and GTP dissociation; it was unexpected that Byr4 appeared to stabilize the GTPbound, active form of Spg1.
byr4&^cdc7	->	spg1	protein	protein	Q10951	P87027	9742395	U	This apparent contradiction with the effect of Byr4 on septation might be explained if Byr4 and Cdc7 bound mutually exclusively to Spg1
cdc7&^byr4	->	spg1	protein	protein	P41892	P87027	9742395	U	This apparent contradiction with the effect of Byr4 on septation might be explained if Byr4 and Cdc7 bound mutually exclusively to Spg1
cdc7	->	cdc16	protein	protein	P41892	P36618	8039497	U	the possibility that p34cdc16 may be a substrate of p120cdc7 or that its activity is regulated through a pathway involving p120cdc7.
cdc2	->	cdc7	protein	protein	P04551	P41892	12546793	U	it is possible that the SPB-associated pool of cdc7p is regulated by cdc2p
rum1	-|	cdc2	protein	protein	P40380	P04551	8521500	S	Paul Nurse, UNIL 16 Sept 2011
cdc2	-|	rum1	protein	protein	P04551	P40380		S	Paul Nurse, UNIL 16 Sept 2011
rum1	-|	cdc2-cdc13	protein	complex	P40380	P04551-P10815	14653990;11527575;15163365,	S	Rum1 is a G1 specific Cdk inhibitor that inactivates the Cdc2/Cdc13 complex in G1 in response to intrinsic signals
cdc2-cdc13	->	plo1	complex	protein	P04551-P10815	P50528	17340144;18793196;11250892	S	Plo1 kinase is activated downstream of MPF
cdc2-cdc13	-|	cdc14-sid1	complex	complex	P04551-P10815	P36589-O14305	17340144	S	MPF opposes the activation of Sid1/Cdc14.
cdc2-cdc13	->	cdc25	complex	protein	P04551-P10815	P06652	12815070;11527575;18301750;11733770	S	MPF activation further boosts Cdc25 and represses Wee1.
cdc2-cdc13	-|	wee1	complex	protein	P04551-P10815	P07527	12815070;11527575;11733770;15163365	S	MPF activation further boosts Cdc25 and represses Wee1.
cdc2-cig1	-|	rum1	complex	protein	P04551-P24865	P40380	9430640	U	The CDK-cyclin complex cdc2-cig1, which is insensitive to p25(rum1)inhibition, seems to be the main kinase that phosphorylates p25(rum1). Phosphorylation of p25(rum1) in S phase and G2 serves as the trigger for p25(rum1) proteolysis.
cdc2-cdc13	-|	rum1	complex	protein	P04551-P10815	P40380	11527575;11733770;15163365	S	Rum1 is phosphorylated by MPF, which promotes the degradation (the phosphorylated form of Rum1 is assumed to degrade very fast)
wee1	-|	cdc2-cdc13	protein	complex	P07527	P04551-P10815	11527575;11733770;15163365	S	Cdc13 Cdc2 kinase is not fully activated at this time, because the Tyr-15 residue of Cdc2 is phosphorylated by two tyrosine kinases, Wee1 and Mik1
cdc25	->	cdc2-cdc13	protein	complex	P06652	P04551-P10815	11527575;11733770	S	In the late G2 phase, this inhibitory phosphate group on Tyr-15 (on MPF) is removed by two tyrosine phosphatases, Cdc25 and Pyp3
plo1	->	spg1	protein	protein	P50528	P87027	17340144;10436027	S	overexpression of plo1+ activates the Spg1 pathway and causes transient Cdc7 recruitment to the SPB and multiple rounds of septation.
dma1	-|	sid4	protein	protein	Q10322	O60187	21131906;21246752	S	the SIN scaffold protein, Sid4, is ubiquitinated in vivo in a Dma1-dependent manner.
dma1	-|	plo1	protein	protein	Q10322	P50528	12479804	S	overexpressing Dma1p reduces SIN signaling. Dma1p seems to function by inhibiting the SIN activator, Plo1p kinase,
sid4&CK1	->	dma1	protein	protein	O60187	Q10322	15062098;20980623	I	Dma1 localizes to the SPB during meiosis and the maintenance of this localization at meiosis II depends on septation initiation network (SIN) scaffold proteins Sid4 and Cdc11.
cdc11	->	dma1	protein	protein	O74473	Q10322	20980623	I	Dma1 localizes to the SPB during meiosis and the maintenance of this localization at meiosis II depends on septation initiation network (SIN) scaffold proteins Sid4 and Cdc11.

sid4	->	cdc11	protein	protein	O60187	O74473	*15062098;11676915*	I	Cdc11p is a phosphoprotein, which becomes hyperphosphorylated during anaphase. It localizes to the spindle pole body at all stages of the cell cycle, in a sid4p-dependent manner, and cdc11p is required for the localization of all the known SIN components, except sid4p, to the SPB. Cdc11p and sid4p can be coimmunoprecipitated from cell extracts.
plo1	->	cdc11	protein	protein	P50528	O74473	*11676915*	U	Which kinase(s) phosphorylate(s) cdc11p? One candidate could be the protein kinase plo1p, since it is thought to act upstream of the SIN
cdc16	->	cdc11	protein	protein	P36618	O74473	*15062098*	I	Cdc16p interacted with the N-terminal 660 amino acids of Cdc11p but not the Cdc11p C-terminal half
spg1	->	cdc11	protein	protein	P87027	O74473	*15062098*	I	In a two-hybrid assay, Spg1p interacted with full-length Cdc11p as well as the Cdc11p N terminus (amino acids 1–551). ...indicating that Cdc11p and Spg1p interact directly.
cdc2	-|	cdc14-sid1	protein	complex	P04551	P36589-O14305	*10775265;12957817*	S	loss of Cdc2-cyclin activity promotes Sid1p-Cdc14p association with the SPB
cdc7	->	cdc14-sid1	protein	complex	P41892	P36589-O14305	*10775265;12957817;11715048*	S	Our localization studies suggest that Sid1p–Cdc14p functions at an intermediate step in the pathway, downstream of Cdc7p and upstream of Sid2p
cdc14-sid1	->	sid2	complex	protein	P36589-O14305	Q09898	*10775265*	S	Our localization studies suggest that Sid1p–Cdc14p functions at an intermediate step in the pathway, downstream of Cdc7p and upstream of Sid2p
cdc2	->	sid1	protein	protein	P04551	O14305	*10775265*	I	We also show that Sid1p localization may be regulated by Cdc2–cyclin activity, suggesting a mechanism that may couple cytokinesis with completion of mitosis.
cdc7	->	sid1	protein	protein	P41892	O14305	*10775265*	S	These results indicate that Cdc14p, Sid1p and Sid2p probably function downstream of Cdc7p, with Cdc14p either functioning upstream of or in conjunction with Sid1p.
cdc14	->	sid1	protein	protein	P36589	O14305	*10775265*	I	These results indicate that Cdc14p, Sid1p and Sid2p probably function downstream of Cdc7p, with Cdc14p either functioning upstream of or in conjunction with Sid1p.
cdc14-sid1	->	sid2-mob1	complex	complex	P36589-O14305	Q09898-O94360	*12957817;11715048*	S	The cdc7 kinase is recruited to the spindle in an spg1-GTP-dependent manner, and activates the sid2-mob1 kinase complex through the sid1-cdc14 kinase
sid2-mob1	->	flp1	complex	protein	Q09898-O94360	Q9P7H1	*12957817*;*19305416*	S	Figure 4
sid2	->	flp1	protein	protein	Q09898	Q9P7H1	18951025;*11715048*;*16085489*	S	sequestered in the nucleolus in interphase; Here we show that the most downstream SIN component, the Ndr-family kinase Sid2, maintains Clp1 in the cytoplasm in late mitosis by phosphorylating Clp1 directly
scd1	->	cdc42	protein	protein	P40995	Q01112	*20175747;12972551;16931912*	S	Fission yeast Cdc42p is activated by a GEF called Scd1p or Ral1p
gef1	->	cdc42	protein	protein	Q09763	Q01112	*20175747;12972551*	S	This study and a parallel study by others establish that Gef1p is another GEF for Cdc42p
plo1	-|	byr4-cdc16	protein	complex	P50528	Q10951-P36618	*19305416*	U	In late mitosis, Plo1 is thought to activate the SIN, possibly by inhibiting Cdc16–Byr4.
slp1	-|	cdc2-cdc13	protein	complex	P78972	P04551-P10815	*15163365*	S	Cdc2/Cdc13 is negatively regulated by four proteins — Slp1, Ste9, Rum1 and Wee1 — and activated by Cdc25.
cdc2-cdc13	->	slp1	complex	protein	P04551-P10815	P78972	*15163365;12779461*	S	Slp1/APC is activated by MPF itself, after a time delay.
ste9	-|	cdc2-cdc13	protein	complex	O13286	P04551-P10815	*9398669;15163365*	S	Cdc2/Cdc13 is negatively regulated by four proteins — Slp1, Ste9, Rum1 and Wee1 — and activated by Cdc25.
cdc2-cdc13	-|	ste9	complex	protein	P04551-P10815	O13286	*10921878*	S	We also show that srw1p is phosphorylated during the cell cycle by the cdc2p–cdc13p protein kinase, and that phosphorylation of srw1p by cdc2p affects the stability of srw1p and inhibits its activity to promote cdc13p turnover.
ste9	-|	ste9	protein	protein	O13286	O13286	*16627999*	I	Together, these data suggest that the AU-rich elements in the 3'UTR of the ste9 mRNA promote its instability and can act autonomously, destabilizing other mRNAs.
ppc89	->	sid4	protein	protein	O60187	Q10218	16775007	I	this observation not only suggests a SIN tethering role for Ppc89 but indicates that the N-terminal 300 amino acids of Sid4 is solely responsible for its essential function in the SIN. This conclusion is consistent with the evidence that Sid4 residues 1–300 contain the docking sites for the checkpoint protein Dma1p, the mitotic kinase Plo1, and also Cdc11, which in turn links to all other SIN components and Cdk1-cyclin B
sid4	->	plo1	protein	protein	O60187	P50528	*15062098;16775007*	I	We find that Sid4p interacts with the SIN activator, Plo1p, in addition to Cdc11p and Dma1p.
cdc16	->	lsk1	protein	protein	P36618	O14098	*15537703*	U	The ability of the lsk1 deletion to abrogate the cdc16–116 phenotype suggests that Lsk1p acts downstream of Cdc16p to promote stability and constriction of the actomyosin ring.
rad24	->	flp1	protein	protein	P42656	Q9P7H1	*16085489*	I	Thus, under a variety of conditions that perturb the cell-division apparatus, Rad24p appears to be involved in the cytoplasmic retention of Clp1p;Rad24p physically interacts with Clp1p;nuclear exclusion of Clp1p under SIN-active conditions depends on Rad24p.

nuc2	-|	spg1	protein	protein	P10505	P87027	*18225957*	I	Nuc2p appears to exert its effects on cytokinesis by modulating the nucleotide state of the Spg1p-GTPase and thereby down regulating the SIN;These experiments established the binding between Cdc7p-3HA and Spg1p-GFP was interrupted in cells overproducing Nuc2p.
nuc2	-|	cdc7	protein	protein	P10505	P41892	*18225957*	I	loss of Nuc2p function leads to persistent localization of SIN components, such as Cdc7p, Sid1p, and Sid2p, even after completion of septation;Nuc2p appears to affect the SPB localization of the SIN kinases but not their stability
nuc2	-|	sid1	protein	protein	P10505	O14305	*18225957*	I	loss of Nuc2p function leads to persistent localization of SIN components, such as Cdc7p, Sid1p, and Sid2p, even after completion of septation;Nuc2p appears to affect the SPB localization of the SIN kinases but not their stability
nuc2	-|	sid2	protein	protein	P10505	Q09898	*18225957*	I	loss of Nuc2p function leads to persistent localization of SIN components, such as Cdc7p, Sid1p, and Sid2p, even after completion of septation;Nuc2p appears to affect the SPB localization of the SIN kinases but not their stability
nuc2	-|	cdc15	protein	protein	P10505	Q09822	*18225957*	I	we tested the effect of Nuc2p overexpression on the localization of the FCH domain protein, Cdc15p, which is essential for actomyosin ring maintenance and septum assembly. As in the case of F-actin, cells were able to assemble Cdc15p rings upon Nuc2p overexpression
nuc2	->	byr4-cdc16	protein	complex	P10505	Q10951-P36618	*18225957*	U	we favour the idea that overproduction of Nuc2p might lead to activation of Byr4p-Cdc16p, thereby to the inability to maintain SIN function and septation.
plo1	->	APC	protein	complex			*11777938*		physical interaction between plo1 and cut 23 (APC8) is crucial for mitotic progression by targeting polo kinase activity toward the APC (mutation of plo1 sites on cut23 leads to metaphase arrest
cdc42	-|	byr4	protein	protein			*8682866*		scd1-null is more sensitive to very mild byr4OP, suggesting that cdc42 stimulates SIN signalling, or inhibits byr4p. The latter is taken for the model, applying Occam's razor.
cdc42	->	pak1	protein	protein			*9858584*		Cdc42 interaction disrupts the intramolecular interactions of Pak1, thereby releasing the kinase from autoinhibition.
pak1	->	orb6	protein	protein			*9636183*		Pak1/Shk1 is required for proper Orb6 intracellular localization (Orb6 kinase acts downstream of a morphogenetic control pathway involving Cdc42 and Pak1/Shk1)
orb6	->	gef1	protein	protein			*19646873*		Orb6 Kinase spatially controls Cdc42 GEF Gef1 localization to the cell tips
pak1	->	pom1	protein	protein			*12764130*		Tea1 (regulator of Pom1), is a potential substrate target of the p21-activated kinase, Shk1; it is directly phosphorylated
pom1	-|	Rga4	protein	protein			*18328707*		Pom1 interacts with Rga4 to exclude it from the cell tips (might not be a direct interaction)
Rga4	-|	cdc42	protein	protein			*18328707; 21849474*		Rga4 is a GAP for cdc42
cdc2-cdc13	->	APC	complex	complex			*10559897*		Cdk needs to be active to activate APC, which in a feedback loop will participate in inactivation of Cdk
APC	-|	cdc2-cdc13	complex	complex			*10559897*		Cdk needs to be active to activate APC, which in a feedback loop will participate in inactivation of Cdk
cdc2-cdc13&	->	SIP	complex	complex			*22119525*		Ppc89 is required for SIP localisation; in cdc25-22 G2 arrested cells, csc1 is not on the SPBs, in prometaphase arrested cells it is on SPBs
cdc2-cdc13	-|	PP	complex	protein			*23333317*		Cdc2-cdc13 dislocalises PP1 from SPBs
SAC	-|	APC	complex	complex			*18556659*		spindle assembly checkpoint proteins inhibit anaphase promoting complex and metaphase-anaphase progression
sid2-mob1	->	nak1					*23394829; 24972934*		Sid2 phosphorylation of Nak1 causes removal of Nak1 from the spindle pole bodies
cdc2-cdc13	->	fin1					*17804403*	I	S. Cerevisiae, The N-terminal half of Fin1 is phosphorylated at multiple sites by the cyclin-dependent kinase Clb5-Cdk1
flp1&sid4	->	cdc11					*23297348*		cdc11 is a flp1 substrate, mutation of clp1 sites to Asp decreases restrictive temp of SIN mutants
cdc2-cdc13&plo1	-|	byr4	protein	protein	P50528	Q10951	*24920823*	S	Our analyses show that Cdk1-mediated phosphoregulation of Byr4 facilitates complete removal of Byr4 from metaphase SPBs in concert with Plo1
