# Supplementary material for: An Extended, Boolean Model of the Septation Initiation Network in S.Pombe Provides Insights into Its Regulation
Source: PLoS One. 2015 Aug 5;10(8):e0134214. doi: 10.1371/journal.pone.0134214 (PMC4526654; doi:10.1371/journal.pone.0134214)
Supplement: S2 Table — The list of interactions comprising the final, optimized model, together on comments justifying their alteration / addition. (PDF) [file pone.0134214.s007.pdf]

<table>
<tr><th>in PKN</th><th>Node 1</th><th>Action</th><th>Node 2</th><th>Node1 type</th><th>Node2 type</th><th>UniProtID</th><th>UniProtID</th><th>PMID (;)</th><th>Class</th><th>Evidence Tag</th><th>Comments</th></tr>
<tr><td>yes-altered</td><td>byr4&cdc16</td><td>-></td><td>byr4-cdc16</td><td>protein</td><td>complex</td><td>Q10951;P36618</td><td>Q10951-P36618</td><td></td><td>S</td><td></td><td>in PKN, functions were assigned independently to byr4 and cdc16. During the optimization we transfered these functions to the complex, and we built in the complex creation mechanism</td></tr>
<tr><td>yes</td><td>byr4-cdc16</td><td>-|</td><td>spg1</td><td>complex</td><td>protein</td><td>Q10951-P36618</td><td>P87027</td><td>10381387;17340144;19305416;9742395;20876564;16691419;18252797;19736319</td><td>S</td><td>Cdc16 and Byr4 form a two component GTPase-activating protein (GAP) for Spg1 that inhibits its activity</td><td></td></tr>
<tr><td>yes-altered</td><td>spg1&cdc11</td><td>-></td><td>cdc7</td><td>protein</td><td>protein</td><td>P87027</td><td>P41892</td><td>19736319;17340144;9742395;9203579;11514436;18252797;16691419;10381387;9420333;</td><td>S</td><td>GTP-bound Spg1 recruits its downstream effector, the Cdc7 kinase</td><td>interaction changed from spg1 activating cdc7 to spg1&cdc11->cdc7</td></tr>
<tr><td>yes</td><td>nak1</td><td>-></td><td>orb6</td><td>protein</td><td>protein</td><td>O75011</td><td>O13310</td><td>20805322;16096637</td><td>S</td><td>Orb6 kinase is activated by the GC kinase Nak1;The exact mechanism by which Nak1 activates Orb6 remains unclear.</td><td></td></tr>
<tr><td>yes</td><td>pmo25</td><td>-></td><td>nak1</td><td>protein</td><td>protein</td><td>Q9P7Q8</td><td>O75011</td><td>20805322;16096637;</td><td>S</td><td>the known dependence of Nak1 activity on Pmo25</td><td></td></tr>
<tr><td>yes</td><td>etd1</td><td>-></td><td>spg1</td><td>protein</td><td>protein</td><td>Q9UTR4</td><td>P87027</td><td>19736319</td><td>S</td><td>Our results suggest that the key function of Etd1 is to cause the hyperactivation of Spg1 that occurs in late anaphase.</td><td></td></tr>
<tr><td>yes-altered</td><td>cdc11&cdc7&^SIP</td><td>-></td><td>sid2</td><td>protein</td><td>protein</td><td>O74473</td><td>Q09898</td><td>10459013;15062098;19736319;10459013;10775265</td><td>S</td><td>A)Cdc11p(488–660) was the smallest tested Cdc11p region that showed a positive interaction with Sid2p. Using a similar strategy, we found that the N terminus but not the kinase domain of Sid2p directed its interaction with Cdc11p. B) SPB-localized Cdc7 then promotes activation of the Sid2 protein kinase</td><td>concatenated cdc7->sid2 and cdc11->sid2, added SIP regulation</td></tr>
<tr><td>yes</td><td>cdc7</td><td>-></td><td>sid2</td><td>protein</td><td>protein</td><td>P41892</td><td>Q09898</td><td>19736319;10459013;10775265</td><td>S</td><td>SPB-localized Cdc7 then promotes activation of the Sid2 protein kinase</td><td></td></tr>
<tr><td>yes</td><td>ras1</td><td>-></td><td>scd1</td><td>protein</td><td>protein</td><td>P08647</td><td>P40995</td><td>8682866;11063680</td><td>U</td><td>ras1 binds and presumably activates the scd1 protein</td><td></td></tr>
<tr><td>yes</td><td>cdc7</td><td>-></td><td>cdc11</td><td>protein</td><td>protein</td><td>P41892</td><td>O74473</td><td>8039497;1527180;12546793</td><td>S</td><td>We demonstrate that mitotic hyperphosphorylation of cdc11p requires the activity of cdc7p</td><td></td></tr>
<tr><td>yes</td><td>cdc7</td><td>-></td><td>pmo25</td><td>protein</td><td>protein</td><td>P41892</td><td>Q9P7Q8</td><td>20876564</td><td>I</td><td>The localization of Pmo25 at the SPBs and the kinase activities of Nak1-Orb6 during interphase are under the control of the Cdc7 and Sid1 SIN kinases, suggesting a functional linkage between SIN and the network for cell morphogenesis</td><td></td></tr>
<tr><td>yes</td><td>cdc14-sid1</td><td>-></td><td>pmo25</td><td>protein</td><td>protein</td><td>O14305</td><td>Q9P7Q8</td><td>20876564</td><td>I</td><td>The localization of Pmo25 at the SPBs and the kinase activities of Nak1-Orb6 during interphase are under the control of the Cdc7 and Sid1 SIN kinases, suggesting a functional linkage between SIN and the network for cell morphogenesis</td><td>sid1 of the PKN became cdc14- sid1 and the cdc14 and sid1 independant functionalities were transfered to the complex</td></tr>
<tr><td>yes</td><td>sid4</td><td>-></td><td>cdc11</td><td>protein</td><td>protein</td><td>O60187</td><td>O74473</td><td>15062098;11676915</td><td>I</td><td>Cdc11p is a phosphoprotein, which becomes hyperphosphorylated during anaphase. It localizes to the spindle pole body at all stages of the cell cycle, in a sid4p-dependent manner, and cdc11p is required for the localization of all the known SIN components, except sid4p, to the SPB. Cdc11p and sid4p can be coimmunoprecipitated from cell extracts.</td><td></td></tr>
<tr><td>yes-altered</td><td>cdc16</td><td>-></td><td>cdc11</td><td>protein</td><td>protein</td><td>P36618</td><td>O74473</td><td>15062098</td><td>I</td><td>Cdc16p interacted with the N-terminal 660 amino acids of Cdc11p but not the Cdc11p C-terminal half</td><td>inversed</td></tr>
<tr><td>yes</td><td>cdc11</td><td>-></td><td>spg1</td><td>protein</td><td>protein</td><td>O74473</td><td>P87027</td><td>15062098</td><td>I</td><td>In a two-hybrid assay, Spg1p interacted with full-length Cdc11p as well as the Cdc11p N terminus (amino acids 1–551). ...indicating that Cdc11p and Spg1p interact directly.</td><td></td></tr>
<tr><td>yes</td><td>sid2-mob1</td><td>-></td><td>flp1</td><td>complex</td><td>protein</td><td>Q09898-O94360</td><td>Q9P7H1</td><td>12957817;19305416</td><td>S</td><td>Figure 4</td><td></td></tr>
<tr><td>yes</td><td>scd1</td><td>-></td><td>cdc42</td><td>protein</td><td>protein</td><td>P40995</td><td>Q01112</td><td>20175747;12972551;16931912</td><td>S</td><td>Fission yeast Cdc42p is activated by a GEF called Scd1p or Ral1p</td><td></td></tr>
<tr><td>yes</td><td>gef1</td><td>-></td><td>cdc42</td><td>protein</td><td>protein</td><td>Q09763</td><td>Q01112</td><td>20175747;12972551</td><td>S</td><td>This study and a parallel study by others establish that Gef1p is another GEF for Cdc42p</td><td></td></tr>
<tr><td>yes</td><td>ppc89</td><td>-></td><td>sid4</td><td>protein</td><td>protein</td><td>O60187</td><td>Q10218</td><td>16775007</td><td>I</td><td>this observation not only suggests a SIN tethering role for Ppc89 but indicates that the N-terminal 300 amino acids of Sid4 is solely responsible for its essential function in the SIN. This conclusion is consistent with the evidence that Sid4 residues 1–300 contain the docking sites for the checkpoint protein Dma1p, the mitotic kinase Plo1, and also Cdc11, which in turn links to all other SIN components and Cdk1-cyclin B</td><td></td></tr>
<tr><td>yes</td><td>cdc42</td><td>-|</td><td>byr4</td><td>protein</td><td>protein</td><td></td><td></td><td>8682866</td><td></td><td>scd1-null is more sensitive to very mild byr4OP, suggesting that cdc42 stimulates SIN signalling, or inhibits byr4p. The latter is taken for the model, applying Occam's razor.</td><td></td></tr>
<tr><td>yes</td><td>cdc42</td><td>-></td><td>pak1</td><td>protein</td><td>protein</td><td></td><td></td><td>9858584</td><td></td><td>Cdc42 interaction disrupts the intramolecular interactions of Pak1, thereby releasing the kinase from autoinhibition.</td><td></td></tr>
<tr><td>yes</td><td>pak1</td><td>-></td><td>orb6</td><td>protein</td><td>protein</td><td></td><td></td><td>9636183</td><td></td><td>Pak1/Shk1 is required for proper Orb6 intracellular localization (Orb6 kinase acts downstream of a morphogenetic control pathway involving Cdc42 and Pak1/Shk1)</td><td></td></tr>
<tr><td>yes</td><td>orb6</td><td>-></td><td>gef1</td><td>protein</td><td>protein</td><td></td><td></td><td>19646873</td><td></td><td>Orb6 Kinase spatially controls Cdc42 GEF Gef1 localization to the cell tips</td><td></td></tr>
<tr><td>yes</td><td>pak1</td><td>-></td><td>pom1</td><td>protein</td><td>protein</td><td></td><td></td><td>12764130</td><td></td><td>Tea1 (regulator of Pom1), is a potential substrate target of the p21-activated kinase, Shk1; it is directly phosphorylated</td><td></td></tr>
<tr><td>yes</td><td>pom1</td><td>-|</td><td>Rga4</td><td>protein</td><td>protein</td><td></td><td></td><td>18328707</td><td></td><td>Pom1 interacts with Rga4 to exclude it from the cell tips (might not be a direct interaction)</td><td></td></tr>
<tr><td>yes</td><td>Rga4</td><td>-|</td><td>cdc42</td><td>protein</td><td>protein</td><td></td><td></td><td>18328707; 21849474</td><td></td><td>Rga4 is a GAP for cdc42</td><td></td></tr>
<tr><td>yes</td><td>sid4</td><td>-></td><td>plo1</td><td>protein</td><td>protein</td><td>O60187</td><td>P50528</td><td>15062098;16775007</td><td>I</td><td>We find that Sid4p interacts with the SIN activator, Plo1p, in addition to Cdc11p and Dma1p.</td><td></td></tr>
<tr><td>yes</td><td>sid4&CK1</td><td>-></td><td>dma1</td><td>protein</td><td>protein</td><td>O60187</td><td>Q10322</td><td>15062098;20980623</td><td>I</td><td>Dma1 localizes to the SPB during meiosis and the maintenance of this localization at meiosis II depends on septation initiation network (SIN) scaffold proteins Sid4 and Cdc11.</td><td></td></tr>
<tr><td>yes-altered</td><td>dma1&sid4</td><td>-|</td><td>plo1</td><td>protein</td><td>protein</td><td>Q10322</td><td>P50528</td><td>12479804</td><td>S</td><td>overexpressing Dma1p reduces SIN signaling. Dma1p seems to function by inhibiting the SIN activator, Plo1p kinase,</td><td>added dependance on sid4</td></tr>
<tr><td>yes</td><td>cdk-H&ppc89</td><td>-></td><td>SIP</td><td>complex</td><td>complex</td><td></td><td></td><td>22119525</td><td></td><td>Ppc89 is required for SIP localisation; in cdc25-22 G2 arrested cells, csc1 is not on the SPBs, in prometaphase arrested cells it is on SPBs</td><td></td></tr>
<tr><td>no</td><td>cdk-0</td><td>-|</td><td>SIP</td><td>complex</td><td>complex</td><td></td><td></td><td>22119525</td><td></td><td>SIP complex dislocation from SPB is linked to Cdc13 degradation (in non-degradable Cdc13 - csc1 on SPBs)</td><td>Adjusted SIP regulation for multi-node CDK</td></tr>
<tr><td>yes-altered</td><td>cdk-0</td><td>-></td><td>flp1</td><td>protein</td><td>protein</td><td>Q9P7H1</td><td>P04551</td><td>16950131</td><td>S</td><td>Cdk1 phosphorylates and inhibits the catalytic activity of the Cdc14 family member, Clp1/Flp1. As Cdk1 activity declines during anaphase progression, Clp1/Flp1 autocatalytically reverses these phosphorylation events to stimulate its own activity.</td><td>adjusted cdc2-|flp1 regulation for multi-node CDK</td></tr>
<tr><td>yes-altered</td><td>cdk-H</td><td>-|</td><td>flp1</td><td>protein</td><td>protein</td><td>Q9P7H1</td><td>P04551</td><td>16950131</td><td>S</td><td>Cdk1 phosphorylates and inhibits the catalytic activity of the Cdc14 family member, Clp1/Flp1. As Cdk1 activity declines during anaphase progression, Clp1/Flp1 autocatalytically reverses these phosphorylation events to stimulate its own activity.</td><td>adjusted cdc2-|flp1 regulation for multi-node CDK</td></tr>
<tr><td>yes-altered</td><td>cdk-L</td><td>-|</td><td>plo1</td><td>complex</td><td>protein</td><td>P04551-P10815</td><td>P50528</td><td>17340144;18793196;11250892</td><td>S</td><td>Plo1 kinase is activated downstream of MPF</td><td>adjusted cdk regulation to plo1 for multi-node CDK</td></tr>
<tr><td>yes-altered</td><td>cdk-H</td><td>-></td><td>plo1</td><td>complex</td><td>protein</td><td>P04551-P10815</td><td>P50528</td><td>17340144;18793196;11250892</td><td>S</td><td>Plo1 kinase is activated downstream of MPF</td><td>adjusted cdk regulation to plo1 for multi-node CDK</td></tr>
<tr><td>yes-altered</td><td>sid2-mob1&^cdk-H</td><td>-></td><td>nak1</td><td></td><td></td><td></td><td></td><td>23394829;   24972934</td><td></td><td>Sid2 phosphorylation of Nak1 causes removal of Nak1 from the spindle pole bodies</td><td></td></tr>
<tr><td>yes</td><td>cdk-H</td><td>-></td><td>fin1</td><td></td><td></td><td></td><td></td><td>17804403</td><td></td><td>S. Cerevisiae, The N-terminal half of Fin1 is phosphorylated at multiple sites by the cyclin-dependent kinase Clb5-Cdk1</td><td></td></tr>
<tr><td>no</td><td>fin1</td><td>-|</td><td>cdc16</td><td></td><td></td><td></td><td></td><td>PREDICTION</td><td></td><td></td><td>predictive link</td></tr>
<tr><td>yes</td><td>flp1&sid4</td><td>-></td><td>cdc11</td><td></td><td></td><td></td><td></td><td>23297348</td><td></td><td>cdc11 is a flp1 substrate, mutation of clp1 sites to Asp decreases restrictive temp of SIN mutants</td><td></td></tr>
<tr><td>yes</td><td>cdc7</td><td>-></td><td>cdc14-sid1</td><td>protein</td><td>complex</td><td>P41892</td><td>P36589-O14305</td><td>10775265;12957817;11715048</td><td>S</td><td>Our localization studies suggest that Sid1p–Cdc14p functions at an intermediate step in the pathway, downstream of Cdc7p and upstream of Sid2p</td><td></td></tr>
<tr><td>yes</td><td>cdk-H</td><td>-|</td><td>cdc14-sid1</td><td>protein</td><td>complex</td><td>P04551</td><td>P36589-O14305</td><td>10775265;12957817</td><td>S</td><td>loss of Cdc2-cyclin activity promotes Sid1p-Cdc14p association with the SPB</td><td>adjusted for multi-node CDK</td></tr>
<tr><td>no</td><td>cdk-L</td><td>-></td><td>byr4</td><td></td><td></td><td></td><td></td><td>10381387</td><td></td><td>co-regulation of SIN by CDK-L and GAP</td><td></td></tr>
<tr><td>yes</td><td>cdk-H&plo1</td><td>-|</td><td>byr4</td><td>protein</td><td>protein</td><td>P50528</td><td>Q10951</td><td>24920823</td><td>S</td><td>Our analyses show that Cdk1-mediated phosphoregulation of Byr4 facilitates complete removal of Byr4 from metaphase SPBs in concert with Plo1</td><td></td></tr>
</table>
